# Dysregulation of septin cytoskeletal organization in the trabecular meshwork contributes to ocular hypertension

**DOI:** 10.1172/jci.insight.179468

**Published:** 2024-12-06

**Authors:** Rupalatha Maddala, Pallavi Gorijavolu, Levi K. Lankford, Nikolai P. Skiba, Pratap Challa, Rakesh K. Singh, K. Saidas Nair, Hélène Choquet, Ponugoti V. Rao

**Affiliations:** 1 Department of Ophthalmology, Duke University School of Medicine, Durham, North Carolina, USA.; 2 Department of Obstetrics and Gynecology, University of Rochester Medical Center, Rochester, New York, USA.; 3 Department of Ophthalmology, UCSF, San Francisco, California, USA.; 4 Kaiser Permanente Northern California (KPNC), Division of Research, Oakland, California, USA.; 5 Department of Pharmacology and Cancer Biology, Duke University School of Medicine, Durham, North Carolina, USA.

**Keywords:** Cell biology, Ophthalmology, Cell migration/adhesion, Cytoskeleton

## Abstract

Ocular hypertension, believed to result partly from increased contractile activity, cell adhesive interactions, and stiffness within the trabecular meshwork (TM), is a major risk factor for glaucoma, a leading cause of blindness. However, the identity of molecular mechanisms governing organization of actomyosin and cell adhesive interactions in the TM remains limited. Based on our previous findings, in which proteomics analyses revealed elevated levels of septins, including septin-9 in human TM cells treated with the ocular hypertensive agent dexamethasone, here, we evaluated the effects of septin-9 overexpression, deficiency, and pharmacological targeting in TM cells. These studies demonstrated a profound impact on actomyosin organization, cell adhesion, contraction, and phagocytosis. Overexpression raised intraocular pressure (IOP) in mice, while inhibition increased cell permeability. In addition, we replicated a significant association between a common variant (rs9038) in *SEPT9* with IOP in the Genetic Epidemiology Research on Adult Healthy and Aging (GERA) cohort. Collectively, these data reveal a link between dysregulated septin cytoskeletal organization in the TM and increased IOP, likely due to enhanced cell contraction, adhesive interactions, and fibrotic activity. This suggests that targeting the septin cytoskeleton could offer a novel approach for lowering IOP in patients with glaucoma.

## Abstract 2

<span>This study highlights the septin cytoskeleton’s importance in intraocular pressure homeostasis and its connection to ocular hypertension pathobiology.</span>

## Graphical abstract

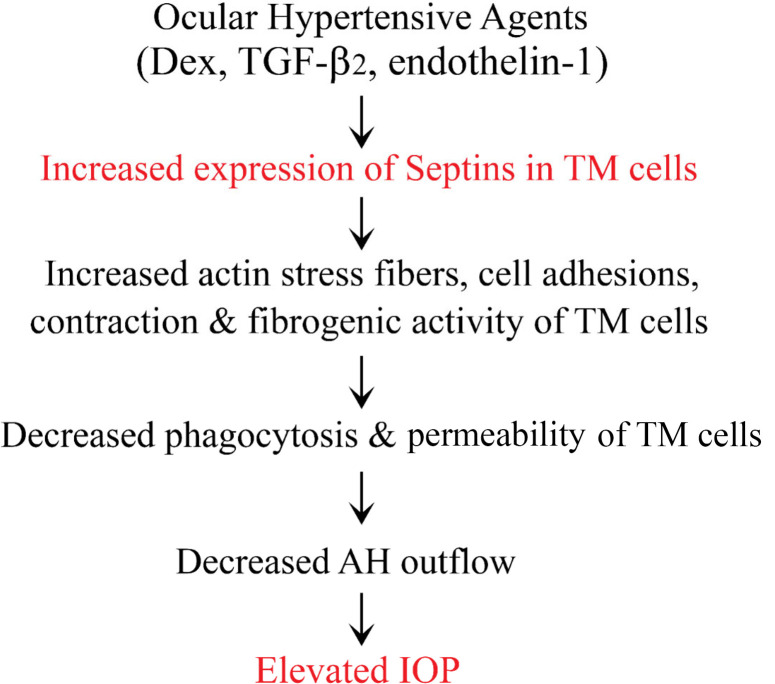

## Introduction

Impaired aqueous humor (AH) drainage through the trabecular meshwork (TM), which results in elevated intraocular pressure (IOP), is a major risk factor for glaucoma, a leading cause of blindness worldwide (1, 2). Lowering IOP is the primary treatment for glaucoma, underscoring the importance of understanding the cellular and molecular mechanisms that regulate AH outflow through the trabecular pathway, which includes both the TM and Schlemm’s canal (SC) (3, 4). Previous studies have shown that various cellular characteristics, such as actomyosin organization, contractility, cell-ECM adhesion, stiffness, and phagocytic properties of TM and SC cells, influence AH outflow and IOP in both animal models and humans (3–12). Notably, Rho kinase inhibitors, recently approved for treating ocular hypertension and glaucoma, target the contractile, fibrotic, and adhesive properties of TM and SC cells (3, 13–16). Despite these advancements, our understanding of the specific cytoskeletal proteins involved in actomyosin contractility and cell adhesion in TM and SC cells, and how these proteins modulate AH outflow and IOP, remains limited.

In our recent study, human TM cells treated with dexamethasone (Dex), a known ocular hypertensive glucocorticoid, exhibited increased levels of various septin proteins, including septin-9 (SEPT9) (17). *SEPT9* and *SEPT7* have been identified as risk loci for IOP and primary open-angle glaucoma, respectively (18, 19). However, our understanding of the roles of septins in TM biology and IOP homeostasis remains very limited (17). Septins are a group of conserved GTP-binding proteins, with mammalian cells expressing 13 different septins organized into 4 subgroups (SEPT2, -3, -6, and -7) based on sequence homology (20–22). Representing the fourth type of cytoskeleton, septins exist as hetero-oligomers that form higher-order structures such as filaments, bundles, gauzes, and rings (23–28). They are distributed to the plasma membrane and interact with the actin cytoskeleton and microtubules, regulating various cellular processes including diffusion barrier activity, permeability, cell adhesion, mechanical properties, phagocytosis, proliferation, and migration (21, 24, 29–31). Mutations and dysregulated expression of septins are linked to various pathologies, highlighting their importance in key physiological functions (32–34). Despite this, the role of septins and the septin cytoskeleton in TM physiology remains unexplored.

Emerging studies suggest that the septin cytoskeleton interacts with the actin cytoskeleton and myosin II (24, 35–41), with their interdependence regulating various cellular activities that could be relevant to IOP homeostasis. Actomyosin organization and cell adhesion are well recognized as modulators of AH outflow and IOP (3, 42). Therefore, this study investigated the role of the septin cytoskeleton, focusing on SEPT9, in TM cells and IOP homeostasis to understand the importance of septin and actin cytoskeletal interactions in the AH outflow pathway. Our results reveal that increased expression of SEPT9 leads to enhanced actin stress fibers, cell adhesive interactions, elevated ECM levels, and contractile responses in TM cells, subsequently elevating IOP in mice.

## Results

### Increased levels of septins in human TM cells induced by ocular hypertensive agents.

**Figure 1:**
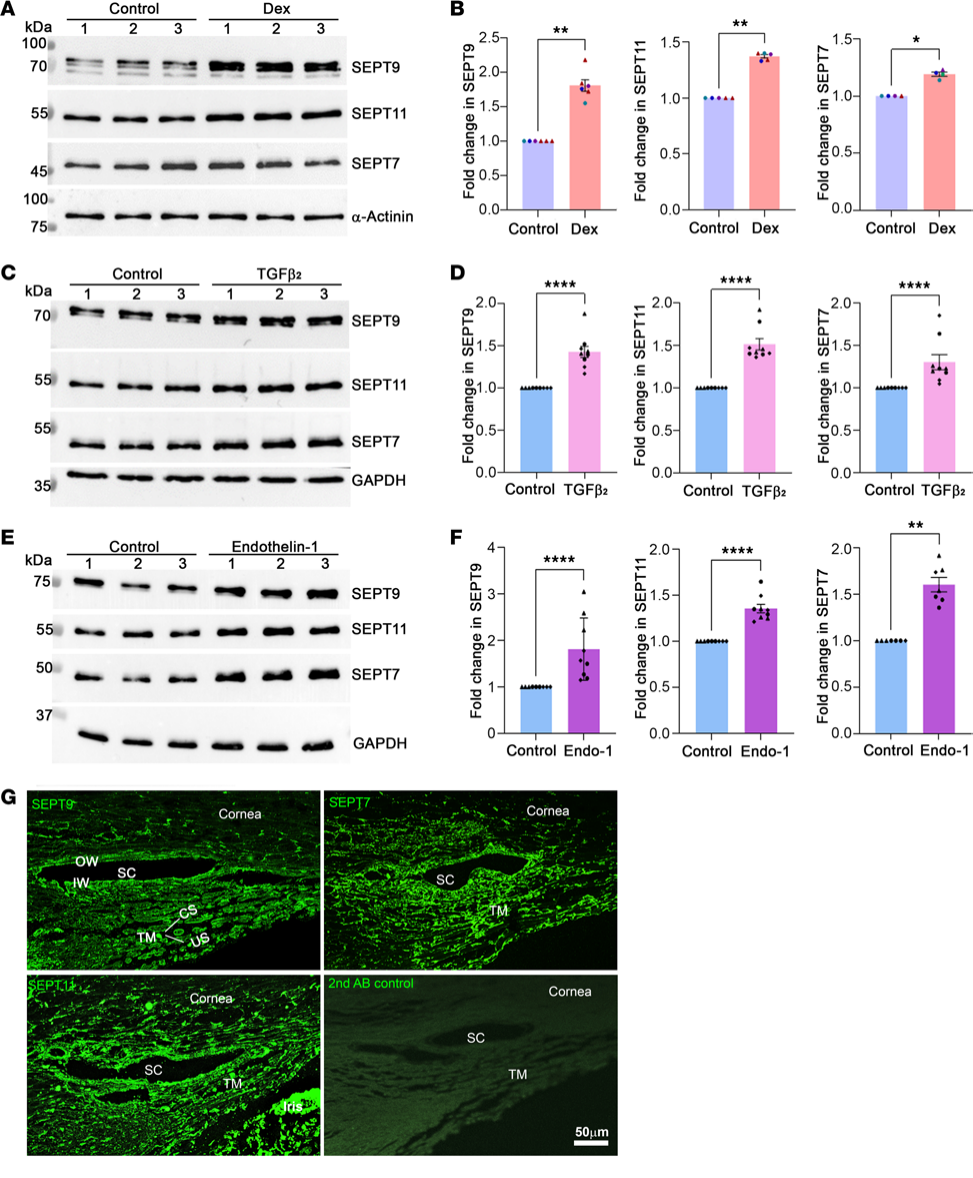
Increased levels of septin-9 and other septins in human trabecular meshwork (TM) cells treated with ocular hypertensive agents, and their distribution in the aqueous humor (AH) outflow pathway. (**A**–**F**) TM cells were treated with Dex (0.5 μM for 7 days), TGF-β2 (10 ng/mL for 24 hours), and endothelin-1 (2 μM for 24 hours). These treatments resulted in significant increases in the levels of SEPT9, SEPT11, and SEPT7 compared with control cells, as determined by immunoblot analyses. Dex effects on septin levels were examined in cytoskeletal fractions, with α-actinin used as a loading control. In contrast, the effects of TGF-β2 and endothelin-1 on septins were assessed in total cell lysates, with GAPDH serving as a loading control. These analyses were conducted using a minimum of 3 different strains of human TM cells, indicated by different colors or shapes in the histograms. Sample size: *n* = 4–6. *P < 0.01, **P < 0.001, ****P < 0.0001 based on Student’s *t* test. Data in **B**, **D**, and **F** are presented as mean fold changes from controls, with error bars representing ±SEM. (**G**) Distribution of SEPT9, SEPT7, and SEPT11 in human AH outflow pathway tissues from 70-year-old donors. The bottom right panel shows background fluorescence from a secondary antibody conjugated with a fluorophore. Scale bar: 50 μm. SC, Schlemm’s canal; US, uveoscleral; CS, corneoscleral; IW, inner wall; OW, outer wall.

Building on our recent findings demonstrating heightened levels of various septins, including SEPT9, in human TM cells subjected to Dex treatment (17) and recognizing septins as integral components of the human TM cell cytoskeletome (43), we initially validated these proteomics-based observations through immunoblot analysis, illustrated in Figure 1. The cytoskeletome fraction isolated from 4 strains of TM cells treated with Dex (0.5 μM for 7 days) exhibited a significant increase in SEPT9, SEPT7, and SEPT11 levels compared with control cells (Figure 1, A and B). α-Actinin 1 was used as a loading control for the cytoskeletal fraction of TM cells to ensure accurate comparison.

Subsequently, we investigated whether this response in septin levels was exclusive to glucocorticoids or if other ocular hypertensive agents similarly influenced TM cells. To explore this, we assessed the impact of TGF-β2 (10 ng/mL, 24 hours) and endothelin-1 (2 μM, 24 hours) on SEPT9 and other septins via immunoblot analyses, using total cell lysates obtained from 3 independent strains of human TM cells. For these total cell lysate samples, glyceraldehyde 3-phosphate dehydrogenase (GAPDH) was used as a loading control. These experiments unveiled significant elevations in SEPT9, SEPT7, and SEPT11 protein levels in both TGF-β2– and endothelin-1–treated cells compared with control cells (Figure 1, C–F). These outcomes parallel the effects observed with Dex treatment, indicating that septins may be common cytoskeletal targets in TM cells regulated by different ocular hypertensive agents. For the agents described, we adjusted treatment durations according to the literature on their known effects on TM cells (3, 7, 12, 43).

Given the absence of information regarding the septin distribution profile within the trabecular pathway, we sought to delineate the distribution patterns of SEPT7, SEPT9, and SEPT11 in the human eye. To address this, we conducted immunofluorescence analysis of paraffin sections of human eyes obtained from 70-year-old donors. Our analysis revealed SEPT7, SEPT9, and SEPT11 distribution throughout both the TM (uveoscleral and corneoscleral regions) and SC (inner wall and outer wall), as depicted in Figure 1G. The tissue sections that were immunostained with only the secondary antibody conjugated to a fluorophore (controls) did not exhibit detectable fluorescence (Figure 1G). These sections were also costained for nuclei using Hoechst, as shown in Supplemental Figure 1; supplemental material available online with this article; https://doi.org/10.1172/jci.insight.179468DS1

Collectively, the expression and distribution of septins in TM cells and tissues of the trabecular pathway indicate that the septin cytoskeleton is a common component of the TM, comparable to microfilaments, microtubules, and intermediate filaments (43).

### Septin and actin cytoskeletal organization exhibit reciprocal interactions in TM cells.

**Figure 2:**
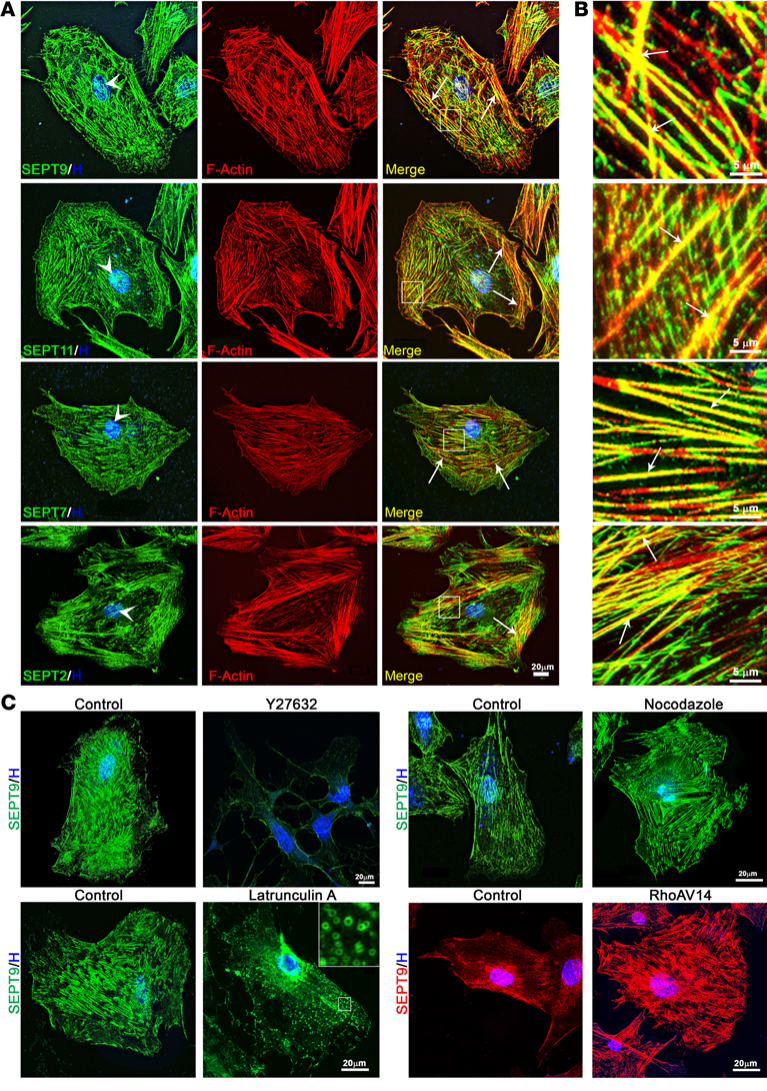
Septin colocalization with actin stress fibers and the impact of cytoskeletal reorganization agents on the septin cytoskeletal architecture in TM cells. (**A** and **B**) These panels show the colocalization of specified septins with F-actin in TM cells. The right-hand images present a higher magnification of the boxed areas in the third panel, illustrating the colocalization of septins with actin stress fibers (arrows). Cell nuclei are stained with Hoechst (blue, arrowheads). (**C**) This panel demonstrates the effects of various cytoskeletal reorganization agents on the septin cytoskeleton (SEPT9 immunostaining) in TM cells. Treatments included the F-actin–depolymerizing agent latrunculin A (2 μM for 5 minutes) that reduces septin filaments, resulting in a ring-like organization of septins (see inset for a magnified view of the boxed area); Rho kinase inhibitor Y2632 (10 μM for 2 hours), an actin stress fiber–suppressing agent that also decreases septin filaments; nocodazole (5 μM for 1 hour), a microtubule-depolymerizing agent that increases actin stress fibers (refer to Supplemental Figure 5) and enhances septin filament formation; and constitutively active RhoA expression, which, similar to nocodazole, increases the formation of septin filaments. Images are representative of a minimum of 3 different TM cell strains. Scale bars: 20 μm (**A** and **C**) and 5 μm (**B**).

The distribution profile of the septin cytoskeleton in TM cells has not been previously documented. Therefore, we determined the distribution patterns of SEPT9, along with other major septins (SEPT7, SEPT2, and SEPT11), in human TM cells. SEPT9 primarily manifested in the membrane-enriched insoluble fraction. Conversely, SEPT11, SEPT7, and SEPT2 were observed in both the soluble and insoluble fractions, as determined by immunoblot analysis (Supplemental Figure 2). Next, for evaluating the cellular distribution pattern of septins, TM cells were cultured on gelatin-coated glass coverslips, and our immunofluorescence analysis revealed a thick, yet short, filamentous arrangement of SEPT9 and other septins throughout the cell body and around the nucleus (blue stain, labeled with Hoechst), with concentrated staining in the cell cortical region relative to the edges (Figure 2A, arrows). Notably, we observed colocalization of septins with actin stress fibers (Figure 2B, merged images, arrows), while their association with focal adhesions was not evident (Supplemental Figure 3), as discerned through confocal fluorescence imaging.

Subsequent experiments involving the disruption of the actin cytoskeletal organization using latrunculin A (2 μM for 5 minutes) and a Rho kinase inhibitor (Y27632, 10 μM for 2 hours) revealed a reduction in septin filaments (Figure 2C) based on immunofluorescence imaging. These agents, as expected, disrupted actin cytoskeletal organization and decreased actin stress fibers in TM cells compared with controls (Supplemental Figure 4). Interestingly, latrunculin A treatment led to septins forming rings in conjunction with decreased filamentous structures (refer to the insert in Figure 2C). In contrast, microtubule depolymerization using nocodazole (5 μM for 1 hour) and the expression of constitutively active RhoA (via an adenoviral vector), which are known to induce actin stress fibers (44, 45), notably induced septin filaments in TM cells (Figure 2C), highlighting a close interaction between the septin and actin cytoskeletons. Supplemental Figures 5 and 6 illustrate the effects of nocodazole and active RhoA on actin stress fibers in TM cells. Specifically, Supplemental Figure 5 shows the induction of actin stress fibers by nocodazole, while Supplemental Figure 6 demonstrates the role of active RhoA in this process. Additionally, these figures reveal the colocalization of microtubules with septin filaments and the depolymerization of microtubule networks induced by nocodazole. Cells treated with latrunculin A and a Rho kinase inhibitor at the specified concentrations were assessed for toxicity and viability using fluorescein diacetate and propidium iodide staining. The results confirmed that the treatments were not toxic, as indicated by the absence of propidium iodide staining (Supplemental Figure 7).

**Figure 3:**
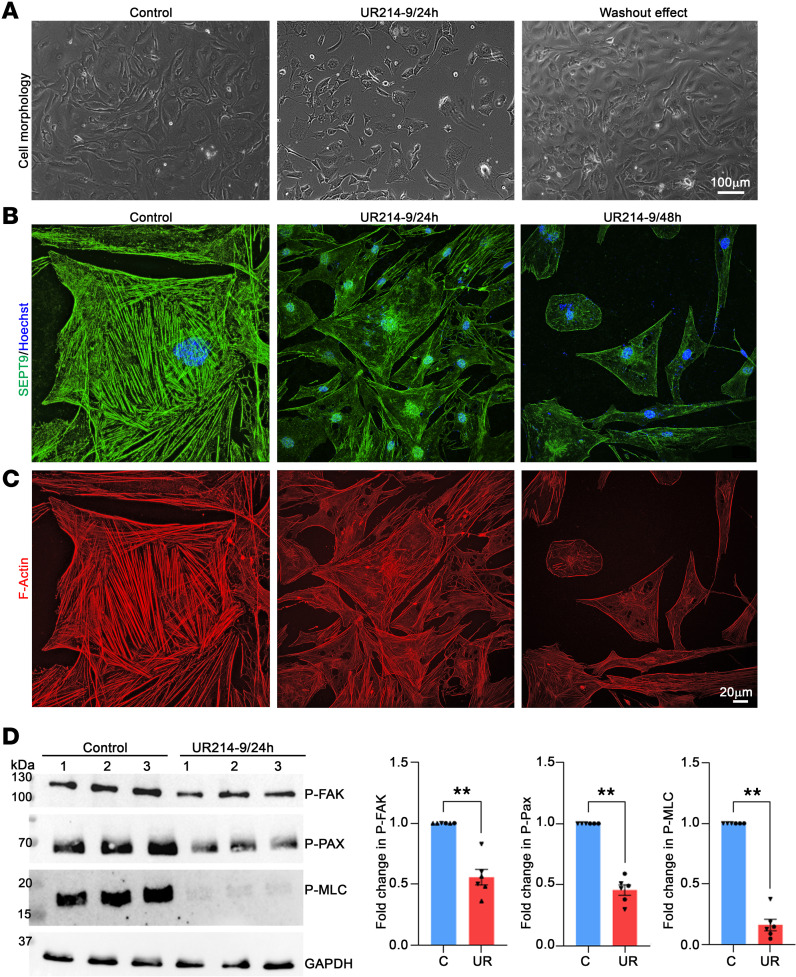
Pharmacological disruption of septin cytoskeletal organization impairs actin cytoskeletal organization, cell adhesion, and myosin II activity in TM cells. (**A**–**D**) TM cells treated with UR214-9 (a septin filament perturbator, 10 μM for 24 and 48 hours) show a time-dependent decrease in septin filaments and actin stress fibers, along with significant reductions in p-paxillin, p-FAK, and p-MLC levels compared with control cells. *n* = 6 (2 strains in triplicate). **P < 0.001 based on Student’s *t* test. Data are presented as mean ± SEM. (**A**) Phase contrast images of TM cells display changes in cell shape induced by UR214-9 treatment, with reversibility observed upon drug washout. Cell nuclei (blue) were stained with Hoechst. GAPDH serves as a loading control. p-MLC blots were developed using urea/glycerol gel electrophoresis as detailed in the Methods section. For the same samples, GAPDH was blotted using a standard SDS-PAGE and transfer protocol, serving as the loading control for p-MLC throughout the study. Scale bars: 100 μm (**A**) and 20 μm (**B** and **C**).

To further delve into the interaction between the septin and actin cytoskeleton in TM cells, we utilized UR214-9, a pharmacological perturbation agent of the septin cytoskeletal dynamics and filament assembly (46, 47), SEPT9-targeting siRNA, and a lentiviral vector (LV) expressing human *SEPT9* (h*SEPT9*) and *eGFP*. Treatment with UR214-9 led to a dose- and time-dependent decrease in septin filamentous cytoskeleton, altering cell morphology (Figure 3). Notably, this effect was reversible (within 24 hours) upon drug withdrawal (Figure 3A, data shown for 10 μM drug treatment over 24 and 48 hours). Under drug-treated conditions, a marked reduction in actin stress fibers was accompanied by a decrease in septin filaments (Figure 3, B and C). Biochemical analyses also demonstrated decreased levels of phospho-paxillin (p-paxillin), phospho–focal adhesion kinase (p-FAK), and phospho–myosin light chain (p-MLC) in UR214-9–treated TM cells compared with controls (Figure 3D). Additionally, UR214-9 decreased septin filaments induced by the constitutively active RhoA along with decreased actin stress fibers (Supplemental Figure 6). The concentrations of UR214-9 utilized in the aforementioned analyses demonstrated no indication of cell toxicity (Supplemental Figure 7). All the fluorescence analyses described above were conducted using at least 3 strains of human TM cells, while the biochemical analyses were performed in triplicate using 2 strains of TM cells.

**Figure 4:**
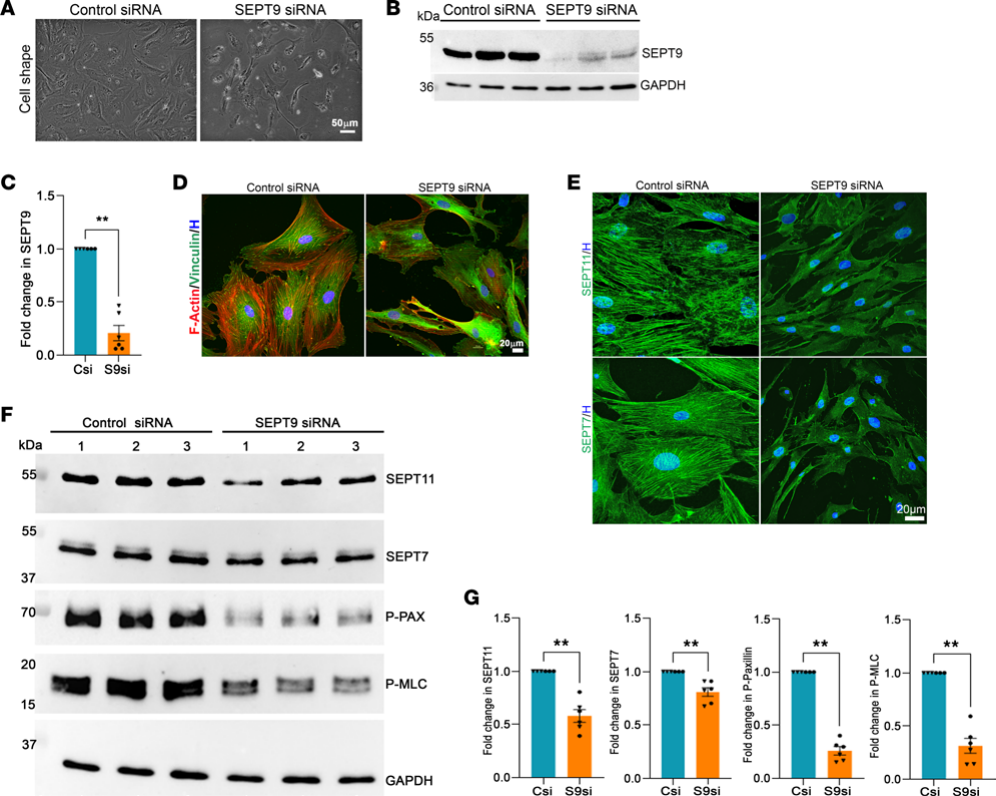
Effects of siRNA-mediated SEPT9 deficiency on cell shape, actin stress fibers, and focal adhesions in TM cells. (**A**–**D**) TM cells treated with SEPT9 siRNA for 72 hours showed a significant reduction (>80%) in SEPT9 protein levels compared with control cells treated with scrambled siRNA. This knockdown of SEPT9 was associated with noticeable changes in cell morphology. Furthermore, SEPT9-deficient TM cells exhibited a decrease in actin stress fibers (**D**, red) and focal adhesions (**D**, vinculin staining, green). (**E**) These cells also showed reduced immunofluorescence for SEPT11 and SEPT7, along with a significant reduction in the levels of SEPT11, SEPT7, p-paxillin, and p-MLC (**F** and **G**) compared with control cells. Cell nuclei were stained with Hoechst (blue). GAPDH was used as a loading control. *n* = 6 (2 strains, in triplicate). **P < 0.001 based on Student’s *t* test. Data are presented as mean ± SEM. Scale bars: 50 μm (**A**) and 20 μm (**D** and **E**). C, control; Csi, control siRNA; S9si, SEPT9 siRNA.

Similar outcomes were observed with *SEPT9* siRNA (60 picomoles for 3 days; Lipofectamine-based transfection), affecting cellular and biochemical attributes as described above (Figure 4, A–G). Suppression of *SEPT9* expression also impacted the levels and distribution of other septins (e.g., SEPT11 and SEPT7 tested), in line with the known formation of hetero-oligomers among septin subtypes (24, 26) (Figure 4, E–G). We performed qRT-PCR analysis to confirm not only the efficacy of siRNA of *SEPT9* in decreasing *SEPT9* expression in TM cells, but also to evaluate its effects on the expression of other septins. *SEPT9* siRNA significantly decreased the expression of *SEPT9* (by 96%), *SEPT7* (by 62%), and *SEPT11* (by 68%) compared with controls (scrambled siRNA treated) (Supplemental Figure 8). For these and other analyses described above, we used triplicates of 2 strains of TM cells. Both immunoblot- and qRT-PCR–based results confirmed altered expression of *SEPT9* having a significant influence on the levels and expression of other septins tested in TM cells.

### SEPT9 induces actin stress fibers, focal adhesions, and contractile characteristics in TM cells.

**Figure 5:**
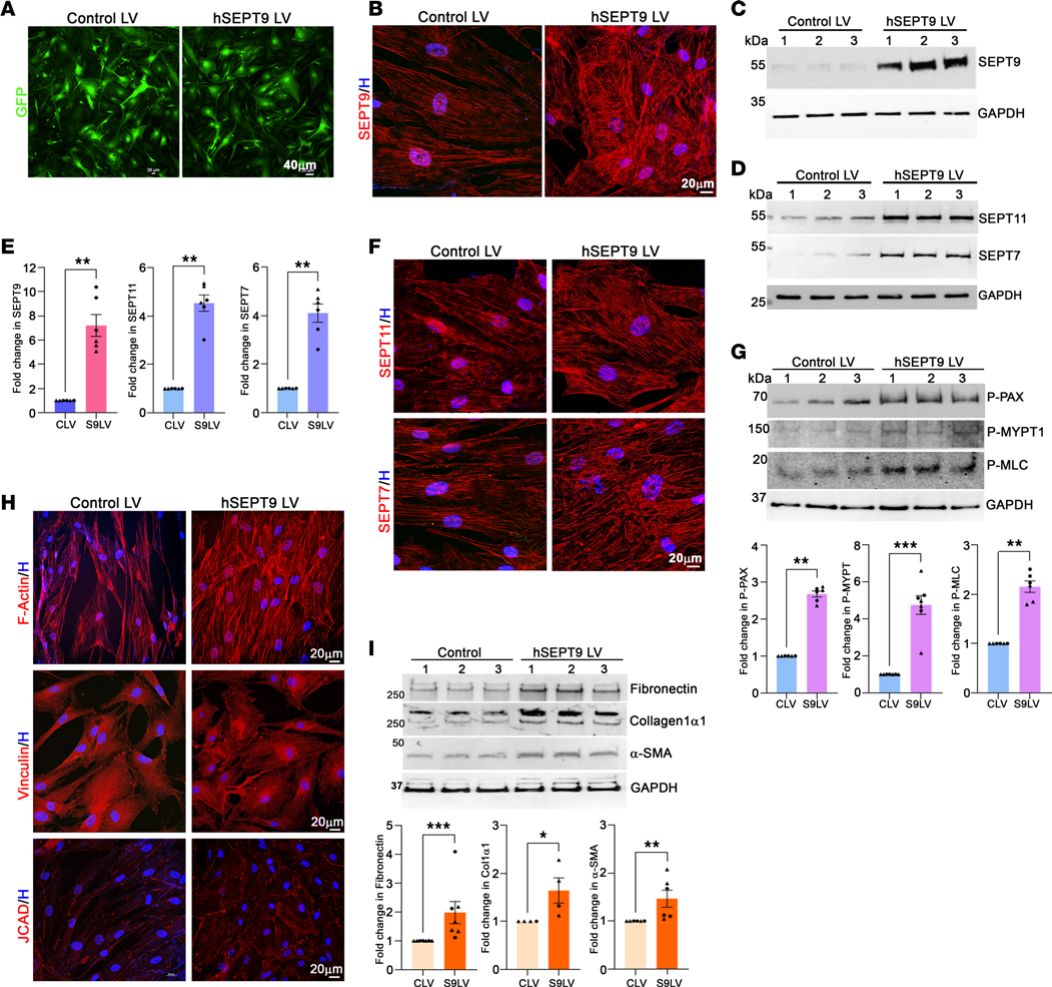
Increased expression of SEPT9 enhances the levels of other septins, actin stress fibers, cell adhesion, and fibrogenic activity in TM cells. (**A**–**F**) Human TM cells were infected with lentiviral vectors expressing either *eGFP* alone (control LV) or h*SEPT9*/*eGFP* (h*SEPT9* LV). The presence of eGFP confirmed successful infection (**A**). Cells expressing h*SEPT9* exhibited significantly higher levels of SEPT9 protein (**C** and **E**) and increased filamentous organization of SEPT9 (**B**) compared with control cells expressing only *eGFP* (**B**). h*SEPT9*-expressing cells also showed significantly elevated levels of SEPT11 and SEPT7 (**D** and **E**), along with increased filamentous organization of these septins (**F**) compared with control cells. (**G**–**I**) TM cells with elevated hSEPT9 levels exhibited enhanced actin stress fiber formation (**H**, F-actin), increased focal adhesions (**H**, vinculin), and cell-cell junction formation (**H**, JCAD). Additionally, these cells showed a significant increase in the levels of p-paxillin (**G**), p-MYPT1 (**G**), p-MLC (**G**), α-SMA (**I**), collagen 1α1 (**I**), and fibronectin (**I**) compared with control cells. Cell nuclei (blue) were stained with Hoechst. GAPDH was used as the loading control. *n* = 6 (2 strains in duplicate or triplicate). *P < 0.05, **P < 0.001, ***P < 0.0001 based on Student’s *t* test. Scale bars: 40 μm (**A**) and 20 μm (**B**, **F**, and **H**). CLV, control lentiviral vector; S9LV, SEPT9 lentiviral vector.

In contrast with the effects of the septin filament assembly perturbation agent (UR214-9) and *SEPT9* siRNA, increased expression of recombinant human SEPT9 via an LV (treated for 8 days) amplified its filamentous organization and protein levels, concurrently elevating SEPT7 and SEPT11 levels compared with cells infected with a control LV expressing *eGFP* (Figure 5, A–E). SEPT11 and SEPT7 also exhibited enhanced filamentous organization in *SEPT9*-overexpressing TM cells (Figure 5F). Moreover, this condition stimulated increased actin stress fibers (phalloidin-TRITC labeling), focal adhesions (vinculin immunostaining), and cell-cell adhesion (junctional protein associated with coronary artery disease [JCAD] immunostaining) compared with control cells (Figure 5H). Additionally, elevated levels of p-paxillin, p-MYPT1 (myosin phosphatase target subunit 1), p-MLC, α-smooth muscle actin (α-SMA), fibronectin, and collagen 1α1 were evident in TM cells expressing recombinant SEPT9 compared with control cells expressing eGFP alone (Figure 5, G and I). These analyses were performed using 2 strains of human TM cells in duplicate or triplicate.

To gain further insight into the influence of SEPT9 on TM cell contractile characteristics, we also evaluated increased expression of *SEPT9* using TM cells (2 strains) expressing recombinant SEPT9 using an LV and collagen gel contraction assay, as described in the Methods. TM cells with increased expression of *SEPT9* compared with control cells revealed a significant increase in collagen gel contraction (based on a decrease in gel diameter), as shown in Supplemental Figure 9, further supporting the influence of septin cytoskeleton on regulation of contractile characteristics of TM cells.

### Identification of septin filament–associated proteins in TM cells.

To gain further insight into the septin filament interaction with other cytoskeletal proteins in TM cells, immunoprecipitation analysis, combined with mass spectrometry using an anti-SEPT9 antibody and TM cell lysates obtained from 2 different strains, revealed a spectrum of coimmunoprecipitated proteins alongside SEPT9. These included prominently other septins (e.g., SEPT2, -7, -10, and -11), as well as a diverse array of cytoskeletal, cytoskeleton-interacting, and cell adhesive proteins (Supplemental Table 1). In addition to the septins, some of the consistently coimmunoprecipitated cytoskeletal and cytoskeleton-interacting proteins included cytoplasmic FMR1-interacting protein, phostensin, adducins, fibronectin type III domain–containing protein 3A, CCN family member 2, palladin, LIM domain and actin-binding protein 1 (EPILIN), synaptojanin-1, α-actinin 4, integrin-linked protein kinase, cytoskeleton-associated protein 5, latent TGF-β–binding protein 2, myosin-9 and 10, different integrins, catenin δ-1, inverted formin-2, drebrin, microtubule-actin cross-linking factor 1, various microtubule binding proteins, filamins, fibrillin, myosin phosphatase Rho-interacting protein (MPRIP), FERM2, EMILIN-1, and others. For a comprehensive list of proteins immunoprecipitated with TM cell strains 1 and 2, refer to Supplemental Tables 2 and 3, respectively. Some of these same proteins identified in this study have been shown to interact and coimmunoprecipitate with septins in other cell types, including myosins (myosin-9 and 10), α-actinin A, phostensin, EPILIN, unconventional myosin-Ic, MPRIP, CD59, LMO7, and others (36, 48, 49). These immunoprecipitation results provide further evidence that septins exist in TM cells as hetero-oligomeric filamentous structures, similar to their known forms in other cell types (24, 49). Additionally, the results demonstrate the interaction of septin filaments with various cytoskeletal proteins.

Collectively, our results from using the septin filament–disrupting agent UR214-9, SEPT9 siRNA, increased SEPT9 expression, and SEPT9 coimmunoprecipitated proteins highlight the interconnected roles of different septins, their filamentous structures, and their interaction with cytoskeletal proteins. These findings reveal that SEPT9 and other septins have a direct impact on actin cytoskeletal organization, actomyosin contraction, focal adhesions, and cell-cell interactions within TM cells. This underscores a reciprocal relationship between the septin and actin cytoskeletons. Notably, elevated SEPT9 expression is associated with increased TM cell contractility, enhanced cell adhesive interactions, ECM production, and a fibrogenic response (Figure 5).

### Increased SEPT9 expression impairs phagocytic activity of TM cells.

**Figure 6:**
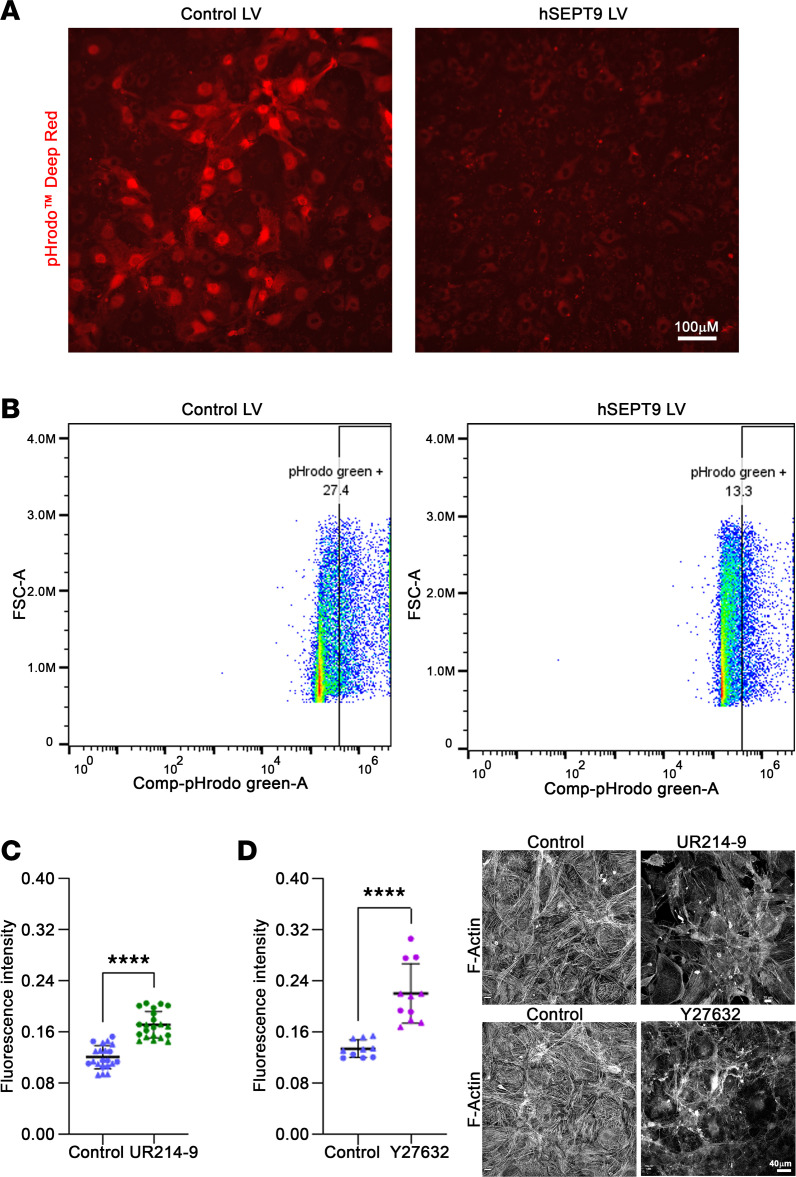
Increased expression of septin-9 impairs TM cell phagocytic activity, and septin filament perturbation enhances fluid diffusion from TM cells grown in Transwells. (**A** and **B**) TM cells expressing h*SEPT9*/*eGFP* exhibit impaired phagocytosis, as evidenced by reduced pHrodo uptake and fluorescence (**A**, red staining) and flow cytometry analysis (**B**), compared with control cells expressing *eGFP* alone. Analyses were performed on equal numbers of cells derived from 3 strains, in triplicate. FSC, forward scatter; LV, lentiviral vector. (**C** and **D**) To assess whether septin cytoskeletal reorganization affects fluid diffusion, TM cells cultured on Transwell inserts for over 1 week were treated with either the septin filament perturbator (10 μM UR214-9 for 18 hours) or the Rho kinase inhibitor (10 μM Y27632 for 2 hours). Following treatment, cells were evaluated for FITC-dextran permeability, as described in the Methods section. Treatment with UR214-9 and Y27632 led to a significant increase in dextran permeability compared with control cells, with Y27632 inducing a more rapid and robust response than UR214-9. Data are presented as mean ± SEM. After treatment, Transwell filters with TM cells were fixed, permeabilized, and stained for F-actin using phalloidin-TRITC. Representative F-actin stained images (**D**, right panel) show a disorganized actin cytoskeleton in cells treated with either the septin perturbator or the Rho kinase inhibitor, compared with vehicle-treated controls. *n* = 4–7 (using 2 strains in multiple). ****P < 0.0001 based on Student’s *t* test. Scale bars: 100 μm (**A**) and 40 μm (**D**).

To unravel the impact of septin cytoskeletal organization on the functional properties of TM cells pertinent to AH outflow, we investigated how increased *SEPT9* expression may influence the phagocytic activity of TM cells, utilizing pHrodo BioParticles conjugates and the previously described LVs. Given the crucial role of actin cytoskeleton organization in regulating phagocytosis (50, 51), a process integral to the filtration function of TM cells (8, 11), we probed the effects of increased *SEPT9* expression on this cellular function. Our investigations revealed a marked and significant reduction in the phagocytic activity of TM cells under increased *SEPT9/eGFP* expression in comparison with control cells expressing *eGFP* alone (Figure 6, A and B). This conclusion was based on the observed pHrodo fluorescence images and flow cytometry analysis. Figure 6, A and B present representative fluorescence images and flow cytometry–based quantification, demonstrating decreased phagocytic activity in TM cells with heightened *SEPT9* expression.

### Pharmacological perturbation of septin cytoskeletal dynamics increases TM cell permeability.

The interplay among TM tissue geometry, actomyosin contractile activity, and the interaction of the actin cytoskeleton with cell adhesive complexes is widely acknowledged to be crucial for the barrier function of the AH outflow pathway (3, 4, 7, 52, 53).

To understand the influence of the septin cytoskeleton on TM cell permeability, which is controlled partly by the organization of actomyosin and cell-cell junctions (31), we conducted an in vitro cell permeability assays utilizing Transwell inserts and FITC-dextran. This assessment aimed to evaluate the impact of the septin filament perturbation agent, UR214-9, in comparison with the Rho kinase inhibitor (Y27632). Notably, cells treated with UR214-9 (10 μM for 18 hours) exhibited a significant increase in FITC-dextran permeability in contrast with control cells (Figure 6C). Meanwhile, the Rho kinase inhibitor (10 μM for 2 hours) induced a more rapid and robust increase in FITC-dextran permeability compared with UR214-9 (Figure 6C). The fluorescence readings presented in Figure 6C were obtained from triplicates across 7 (totaling 21 readings) and 4 Transwells (12 readings) for UR214-9 and Y27632 treatments, respectively, and are represented as mean ± SEM. We used 2 TM cell strains for this analysis.

Notably, the effects of both UR214-9 and Y27632 on TM cell permeability appeared to coincide with the disorganization of actin stress fibers, as evidenced by the phalloidin-TRITC fluorescence images of TM cells grown on the Transwell filters (Figure 6D, right panel). These findings underscore the influence of the septin cytoskeleton and actin cytoskeleton on TM cell permeability.

### Increased expression of SEPT9 elevates IOP in mice.

**Figure 7:**
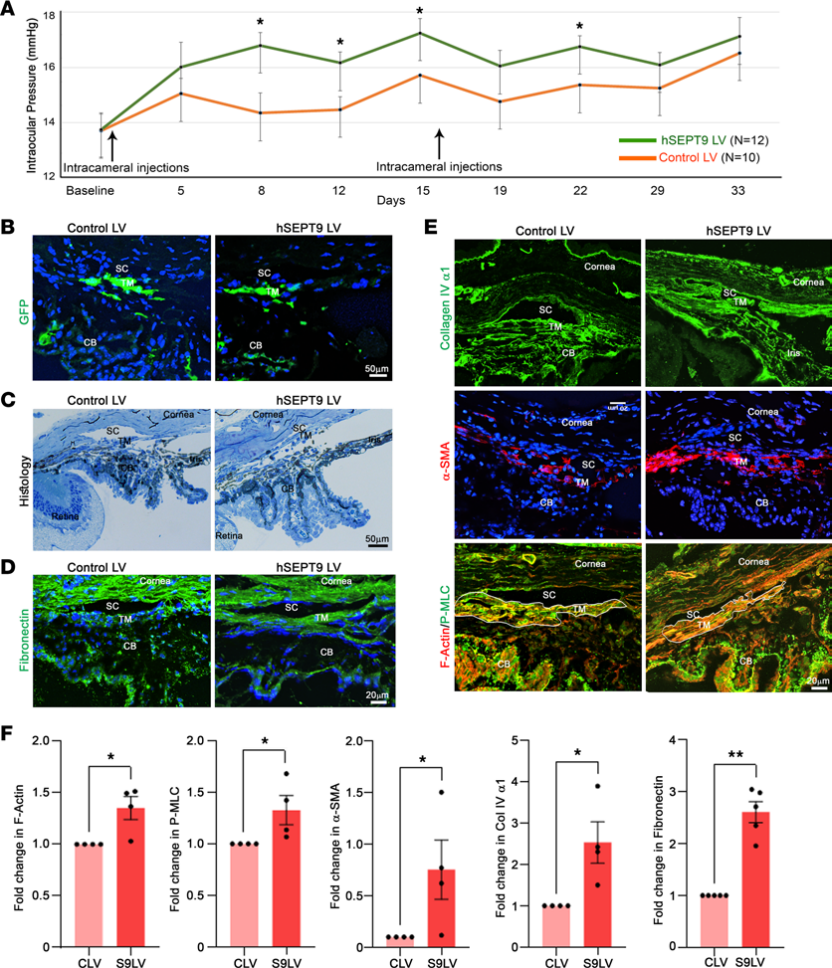
Elevation of IOP in mice expressing increased levels of human SEPT9 in the AH outflow pathway. (**A**) Adult wild-type mice were bilaterally injected with either h*SEPT9*/*eGFP*-LV or control/*eGFP*-LV. Mice injected with h*SEPT9*/*eGFP*-LV exhibited consistent and significant IOP elevation compared with those injected with control/*eGFP*-LV. Viral vector injections were repeated after 2 weeks. *n* = 10–12 eyes. *P < 0.05 based on the nonparametric Wilcoxon’s rank-sum test. Data are presented as mean ± SEM; mouse experiments were repeated twice. (**B**) Expression of recombinant protein in the AH outflow pathway was confirmed in mice injected with the viral vectors through eGFP fluorescence detection. (**C**) Light microscopy–based histological examination of the AH outflow pathway tissues revealed no notable differences (except the SC lumen area) between the h*SEPT9*/*eGFP*-expressing and control (*eGFP*-expressing) mice. (**D**–**F**) Immunofluorescent staining of the AH outflow pathway tissues (especially the TM) showed increased levels of fibronectin, collagen IV, α-SMA, p-MLC, and F-actin in mice expressing h*SEPT9*/*eGFP* compared with control specimens. Representative images are shown (*n* = 4). (**F**) Quantification of immunofluorescence in the trabecular pathway (TM and inner wall of SC) revealed a significant increase in F-actin, p-MLC, α-SMA, collagen IV α1, and fibronectin in h*SEPT9*/*eGFP*-expressing mice compared with control mice. Tissue sections were stained with Hoechst (nuclei staining in blue). Representative ROI tracing used for the TM and inner wall of SC fluorescence quantification is shown in **E** (see the F-actin panel for reference). *n* = 4. *P < 0.05, **P < 0.01 based on Student’s *t* test. Scale bars: 50 μm (**B** and **C**) and 20 μm (**D** and **E**). TM, trabecular meshwork; SC, Schlemm’s canal; CB, ciliary body; LV, lentiviral vector; CLV, control lentiviral vector; S9LV, *SEPT9*-expressing lentiviral vector.

To investigate whether dysregulated septin cytoskeletal organization due to increased *SEPT9* expression influences IOP, we performed intracameral injections of an LV expressing h*SEPT9* in the anterior chamber of the eyes of adult mice and monitored IOP changes in a time-dependent manner. We used C57BL/6J mice (male and female) aged 4 months. The mice received LV injections twice during the study, with the first injection after recording baseline IOP and the second injection 2 weeks later. Expression of the LV-delivered gene was confirmed by imaging eGFP fluorescence (Figure 7). IOP values (diurnal) were consistently higher in mice expressing h*SEPT9*, with the recoded IOP significantly elevated compared with *eGFP*-expressing control mice (Figure 7). As shown in Figure 7A, although IOP elevation found in h*SEPT9*-expressing mice was moderate, this in vivo mouse study provides evidence for the direct influence of increased SEPT9 expression on IOP. IOPs were compared between the control mice and those expressing h*SEPT9* at the indicated time points. In future studies, it will be important to investigate how increased coexpression of different septins affects IOP, given that septins function as hetero-oligomeric filaments (49).

Histological examination of the trabecular pathway in h*SEPT9*-expressing mice and control mice, using light microscopy, revealed no overt differences between the 2 groups. However, morphometric analysis showed a significant reduction in the SC lumen area in h*SEPT9*-expressing specimens compared with controls (Supplemental Figure 10). In contrast, transmission electron microscopy (TEM) revealed subtle differences. Quantitative assessment of TEM images indicated a decrease in the number of large vacuoles and an increase in small vacuoles in SC cells. Additionally, visual evaluation suggested that the TM tissue appeared more compressed or firm in h*SEPT9*-expressing specimens than in controls (Supplemental Figure 10). Although the changes in large- and small-vacuole numbers were not statistically significant, the observed trends of decreased large vacuoles, increased small vacuoles, and a significant reduction in SC lumen area suggest that increased *SEPT9* expression may influence the geometry of both the TM and SC.

### Evidence for increased fibrogenic activity due to increased SEPT9 expression in the TM.

To understand how increased *SEPT9* expression may lead to elevated IOP, we examined tissue sections derived from the iridocorneal angle of eyes enucleated from mice used in the IOP study described above. The tissue cryosections derived from the mice injected with LVs for approximately 5 weeks were immunostained for α-SMA, fibronectin, collagen IV α1, and p-MLC, and stained for F-actin. Based on the immunofluorescence quantification of the TM and inner wall of SC regions derived from 5 individual eye specimens, there was a significant increase in staining (shown as fold change from the controls) for fibronectin, F-actin, p-MLC, α-SMA, and collagen IV α1 in samples derived from mice expressing h*SEPT9*/eGFP compared with *eGFP*-expressing control mice (Figure 7, D–F). Additionally, we immunostained the tissue specimens for the inflammation markers Iba-1 and F4/80. Neither the control specimens nor the h*SEPT9*-expressing specimens from the LV injections showed positive staining for these markers. Figure 7E (F-actin labeling) presents a representative tracing of the region of interest (ROI) used for the TM/inner wall of the SC, which was employed to quantify the immunofluorescence changes in the proteins mentioned above (Figure 7F).

These findings, along with the SEPT9-induced changes in TM cell actin stress fibers, collagen gel contraction, focal adhesions, and phosphorylation of paxillin, MYPT1 and MLC, as shown in Figure 5, G–I, collectively suggest that the increased contractile activity, ECM accumulation, and fibrogenic response (evidenced by increased levels of α-SMA and ECM) in the trabecular pathway are driven by elevated *SEPT9* expression and septin filament formation likely contributing to increased resistance to AH outflow, leading to elevated IOP.

### Replication of SEPT9 association with IOP in the GERA cohort.

Given that the *SEPT9* genetic variant rs9038 was previously reported to be associated with IOP (18), we sought to replicate this association in an independent cohort, the Genetic Epidemiology Research on Adult Healthy and Aging (GERA) cohort. We found a significant association between *SEPT9* genetic variant rs9038 and IOP (*P* = 5.3 × 10^–7^), as illustrated in a regional plot (Supplemental Figure 11). Thus, this independent replication of a common variant in *SEPT9* with IOP in addition to the above-mentioned functional characterization evidence support the contribution of *SEPT9* to the mechanisms underlying IOP regulation.

## Discussion

**Figure 8:**
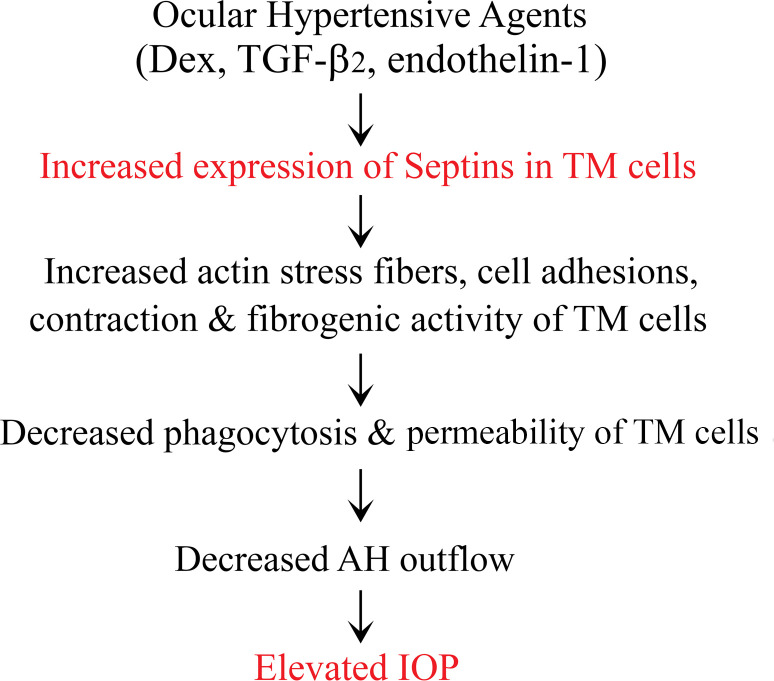
A schematic depiction of the effects of increased expression of septins on the TM cell functional characteristics leading to elevated IOP. Presentation summarizes the effects of increased expression of septins on the TM cell functional characteristics leading to elevated IOP.

This study is the first, to our knowledge, to demonstrate a direct role for the septin cytoskeleton in modulating IOP in live mice. Our findings show that treating TM cells with various ocular hypertensive agents leads to an elevation in septins, which are key components of the TM cytoskeleton (43). Furthermore, we reveal the interaction between septins and the actin cytoskeleton, highlighting a reciprocal influence on the organization of the septin cytoskeleton and actomyosin networks. This coordinated interaction impacts contractile, cell adhesive, and fibrogenic activities within TM cells. These insights collectively illustrate how dysregulated septin cytoskeletal dynamics can disrupt IOP homeostasis. This disruption occurs by impairing TM cell phagocytic activity, enhancing fibrogenic activity, and potentially altering biomechanical properties through increased actomyosin contractility, cell adhesive interactions, and ECM production. Additionally, we provide further evidence supporting the association between the SEPT9 genetic variant rs9038 and IOP. Figure 8 presents a schematic summary of the effects of the septin cytoskeleton on TM cells and IOP.

Our initial insights into the plausible role of septins and their cytoskeletal interactions in TM cell physiology emerged from our prior studies on Dex’s effects on the TM cell nuclear proteome profile (17). In the current study, we extended these prior findings, demonstrating the elevation of different septins in TM cells by not only glucocorticoids, but also other ocular hypertensive agents like TGF-β2 and endothelin-1.

Our investigations into septin colocalization with the actin cytoskeleton and cell adhesion proteins, as well as the effects of actin cytoskeletal modulators like latrunculin A, Rho kinase inhibitor, nocodazole, active RhoA GTPase, and a septin cytoskeletal inhibitor in TM cells, revealed interactions of various septins with the actin cytoskeleton. Notably, disruption of the septin filament assembly using UR214-9 and SEPT9 deficiency altered cell morphology in association with decreased actin stress fibers, suggesting mutual interactions between the septin cytoskeletal organization and actin stress fiber formation in TM cells and their collective impact on cell contractile and adhesive characteristics. Moreover, increased *SEPT9* expression induced actin stress fibers, focal adhesions, cell-cell adhesion, and reduced phagocytic activity, essential for TM filtration function (11). Additionally, pharmacological disruption of septin cytoskeletal assembly increased TM cell permeability, potentially due to disrupted actomyosin organization, and cell-cell junctions. The changes in TM cell characteristics, including changes in actin stress fibers, cell adhesion, contraction, and ECM accumulation induced by elevated septin levels and filament formation, are likely to increase resistance to AH outflow, leading to elevated IOP (3). Like findings in other cell types (36, 40, 54), changes in SEPT9 expression in TM cells influenced the expression and protein levels of other septin subtypes. Therefore, the observed changes in both TM cells and in live mice with altered SEPT9 expression are likely driven by dysregulated organization of the hetero-oligomeric septin cytoskeleton, composed of different septin subtypes.

While septin distribution in the focal adhesions of TM cells is not readily apparent, manipulating septin cytoskeletal organization either by using SEPT9 siRNA or through SEPT9 overexpression has demonstrated its role in activating focal adhesion formation in TM cells. This activation appears to occur indirectly, likely mediated by septin’s influence on the formation and stability of actin stress fibers (55). Furthermore, in TM cells, immunoprecipitation of SEPT9 not only captured other septin subtypes, but also a variety of proteins associated with cytoskeletal interactions, cell adhesion, integrin-linked kinase, actin cross-linking proteins, and myosin II, key components involved in cell contraction and adhesion. Recent studies have also revealed interactions between septins and certain focal adhesion proteins (48, 56), as well as the activation of Rho GTPase signaling and focal adhesion formation in different cell types by septins (56–58). Notably, septins and their filaments have been implicated in the regulation of mechanotransduction and matrix stiffness, coordinating with actomyosin organization and stability (29). These processes are known to affect AH outflow and IOP (9, 10).

Our results from live mice with increased h*SEPT9* expression show a significant elevation in IOP, accompanied by higher levels of α-SMA, p-MLC, F-actin, fibronectin, and collagen. Both cell culture experiments and live mouse studies indicate that heightened formation of septin cytoskeletal filaments drives increased actin stress fibers, contractile activity, cell adhesion, and fibrogenic responses. This cascade results in decreased phagocytic activity and may alter both the biomechanical and histological properties of the TM and SC, contributing to increased resistance to AH outflow and, consequently, elevated IOP (Figure 7). In Figure 2C, we demonstrate that constitutively active RhoA, a known potent inducer of actin stress fibers (3, 45), triggers the formation of septin filaments in TM cells. Our previous study found elevated RhoA expression in the trabecular pathway of rats, leading to increased IOP, associated with enhances in actomyosin-driven contraction, α-SMA expression, and fibrogenic activity (15). This finding is consistent with our current study, where heightened *SEPT9* expression in mice correlates with an increase in IOP (Figure 7). Given the heteromeric interactions among the 4 septin subtypes, future research should consider investigating the effects of coexpressing different septin subtypes on IOP. This would provide a broader understanding of septin’s role, beyond the moderate IOP elevation observed with SEPT9 alone in this study.

Furthermore, our findings suggest that the septin cytoskeleton within the trabecular pathway is a promising target for IOP reduction, similar to actomyosin. Forchlorfenuron-related compounds have shown the ability to modulate septin cytoskeletal dynamics and organization in various cell types (47, 59). The effects of UR214-9 on TM cell morphology, actin stress fibers, cell adhesion, and permeability further support the potential of pharmacologically targeting the septin cytoskeleton in the TM as a treatment for ocular hypertension. This approach may offer an alternative strategy for indirectly influencing actomyosin networks and contraction by disrupting the septin cytoskeleton. This is particularly important given that direct targeting of actomyosin networks in the TM through Rho kinase inhibitors, while effective at reducing IOP in glaucoma patients (3, 13, 14), is associated with multiple adverse effects (60). However, the topical effects of UR214-9 were not explored in the current study due to a lack of established diffusion through the cornea and unknown pharmacokinetics in the aqueous humor. Future preclinical studies targeting the septin cytoskeleton in the trabecular pathway, both as a standalone treatment and in combination with Rho kinase inhibitors, could lead to substantial outcomes. This approach will help to further elucidate the translational potential and efficacy of septin cytoskeleton disruptors in lowering IOP.

In summary, this study highlights the upregulation of SEPT9 and other septins in TM cells exposed to various ocular hypertensive agents. This upregulation influences the septin cytoskeletal organization, affecting key TM cell functions such as actomyosin contractility, cell adhesion, fibrogenesis, phagocytosis, and permeability, ultimately impacting IOP. These findings, along with the association between a SEPT9 genetic variant and IOP across multiple cohorts, including the GERA cohort (18), emphasize the crucial role of the septin cytoskeleton in IOP regulation. Notably, dysregulation of septin cytoskeletal organization may contribute to elevated IOP, identifying it as a potential target for glaucoma treatment.

## Methods

### Sex as a biological variable

While sex-specific changes in TM cell biology and AH outflow related to IOP remain unknown, this research accounts for sex as a biological variable. Consequently, both male and female subjects were included in all mouse and human studies described herein.

### TM cell culture and treatments

Human TM cells were used for all cell culture studies, which included a minimum of 2 strains and were conducted using technical duplicates or triplicates. The TM cells were cultured from human male and female donor corneal rims, as previously described (43). We utilized primary cell cultures from individuals aged 18 to 72 years (passage 3 to 6), following the consensus recommendations provided by Keller et al. (61). TM cells were cultured in Dulbecco’s modified Eagle’s medium (DMEM) containing low glucose with 10% heat-inactivated fetal bovine serum (FBS), 100 U/mL penicillin, 100 μg/mL streptomycin, and 2 mM L-glutamine at 37°C, in a sterile, humidified incubator under 5% CO_2_.

Upon reaching 80% confluence, TM cells were maintained in medium containing 2% FBS for 2 hours before treatment with 0.5 μM Dex (D8893, Sigma-Aldrich) for 7 days, with media changes every other day consisting of fresh 2% FBS–containing media with Dex added as previously described (17). Control cells were treated accordingly with the respective alcohol vehicle, and cells were harvested for the cytoskeletal fraction, following our previously established protocol (43). For treatment with TGF-β2 (10 ng/mL; H8666, MilliporeSigma), endothelin-1 (2 μM; E7764, MilliporeSigma), and the septin cytoskeletal disruptor UR214-9 (10 μM; supplied in-house), cells were maintained overnight in cell culture media containing 1% FBS prior to treatment. After the treatment period, cells were imaged using a Nikon Eclipse Ts2R microscope, and then harvested for total lysate preparation in 8 M urea buffer for use in immunoblotting analysis.

TM cells grown on gelatin-coated glass coverslips to 80% confluence in complete media were serum starved to 1% serum. After 2 hours, these cells were treated for specified durations with various agents, including 5 μM Rho kinase inhibitor Y27632 (Mitsubishi Tanabe Pharma), 2 μM latrunculin A (428021, MilliporeSigma), 5 μM nocodazole (74151, Fluka Chemie/MilliporeSigma), and 10 μM UR214-9.

To evaluate the effects of constitutively active RhoA, cells maintained in 10% FBS–containing media were infected with replication-defective recombinant adenoviral vectors encoding either eGFP alone (control) or constitutively active RhoA (RhoAV14; multiplicity of infection [MOI] = 30) tagged with eGFP, as previously described (3). Following 48 hours of infection, when 70%–80% of the cells expressed eGFP fluorescence, the cells were starved for 24 hours before fixation for analysis. For testing the effects of UR214-9 on RhoA-induced septin filament and actin cytoskeletal organization, the cells were infected with RhoA-expressing viral vector for 24 hours, rinsed with media, and treated with 10 μM UR214-9 for 24 hours and fixed for the described analyses along with the respective controls treated with eGFP-expressing viral vector.

To test the effects of human SEPT9 siRNA, TM cells grown in 6-well cell culture plates or on gelatin-coated glass coverslips (60%–80% confluent) were treated with 60 picomoles of SEPT9 siRNA (ON-TARGETplus human SEPT9 siRNA-SMARTpool) or scrambled siRNA (ON-TARGETplus non-targeting Pool, from Horizon Discovery Biosciences Limited), preincubated for 20 minutes along with Opti-MEM (Gibco) containing Lipofectamine RNA iMAX transfection reagent (Thermo Fisher Scientific). After 72 hours of treatment, cells were harvested for the described analyses. To determine the effects of increased expression of *SEPT9*, TM cells (60%–80% confluent) were infected with the control LV (25 MOI) expressing *eGFP* alone or LV-h*SEPT9* expressing human *SEPT9* and *eGFP*. Cells were monitored regularly for eGFP expression. After 8 days of LV infection, cells were imaged for eGFP fluorescence using a Nikon Eclipse Ts2R microscope, and thereafter harvested for the described analyses.

### Immunoblot analysis

Following the various treatments described above, cells were rinsed with 1× cold PBS and treated with 10% ice-cold trichloroacetic acid containing 0.5 M DTT (43). Precipitates obtained after centrifugation at 800*g* were suspended in an 8 M urea buffer containing 20 mM Tris, 23 mM glycine, 10 mM DTT, and saturated sucrose, and briefly sonicated. Protein concentration was determined using the Micro BCA Protein Assay Kit (23235, Thermo Fisher Scientific). For isolation of cytosolic and membrane-enriched fractions from TM cells maintained in normal growth media, cells were scraped and suspended in a hypotonic buffer (17), and were sonicated and centrifuged at 800*g*; the resultant supernatant was ultracentrifuged at 100,000*g* for 1 hour. Supernatants were collected as cytosolic fractions and the pellets were washed again, and the resultant membrane-enriched pellets were suspended in 8 M urea buffer. Protein concentrations in both the cytosolic and membrane-enriched fractions were determined as described above.

For immunoblotting, equal amounts of the protein were mixed with 2× Laemmli sample buffer and separated by 8% or 10% SDS-PAGE, followed by transfer to nitrocellulose membranes. Membranes were blocked for 2 hours at room temperature in Tris-buffered saline (TBS) containing 5% (wt/vol) nonfat dry milk and 0.1% Tween 20 and subsequently probed overnight at 4°C with respective primary antibodies (Supplemental Table 4). Membranes were washed with TBS buffer containing 1% Tween 20 and incubated with appropriate secondary antibodies (Supplemental Table 4) for 2 hours at room temperature. Immunoblots were developed using SuperSignal West Femto Maximum sensitivity substrate (Thermo Fisher Scientific), followed by scanning and analysis using ChemiDoc Touch imaging and Image Lab Touch Software (Bio-Rad Laboratories), respectively.

To assess changes in p-MLC levels, the acid-precipitated protein samples, as described above, were separated by urea/glycerol gel electrophoresis and transferred onto nitrocellulose membranes, following our previously published protocol (5). The membranes were then immunoblotted using a polyclonal anti–p-MLC antibody and the appropriate secondary antibody (Supplemental Table 4). The same protein samples used for p-MLC analysis were also immunoblotted for GAPDH, using standard SDS-PAGE and transfer protocols described above, to serve as a loading control for the p-MLC blots.

### Immunofluorescence, imaging, and quantification

Cells subjected to the aforementioned treatments were fixed with 4% paraformaldehyde, washed with cytoskeletal buffer (43), permeabilized for 10 minutes with 0.5% Triton X-100 in PBS, and blocked with a serum-containing buffer (10% FBS in PBS with 0.02% sodium azide). Immunostaining was carried out overnight in serum-containing buffer with 0.2% saponin and the appropriate primary antibodies (Supplemental Table 4), followed by incubation with appropriate secondary antibodies conjugated with Alexa Fluor 488 or 568 for 2 hours at room temperature. For F-actin staining, permeabilized TM cells were incubated with either phalloidin-TRITC (Sigma-Aldrich) or phalloidin conjugated with Alexa Fluor 647 (Thermo Fisher Scientific). Cell nuclei were counterstained with Hoechst (Hoechst 33258, Thermo Fisher Scientific). Finally, coverslips were mounted onto glass slides using Shandon Immu-Mount (Thermo Fisher Scientific), and images were captured using a Nikon Eclipse 90i confocal laser-scanning microscope.

For immunostaining of SEPT9, SEPT7, and SEPT11 in human eye globe sections, paraffin-embedded sections were deparaffinized in xylene and rehydrated, as previously described (15). The tissue sections were blocked for 10 minutes in a humidified chamber with a medical background Sniper reducing solution (Biocare Medical), prior to incubation with rabbit anti-SEPT9, -SEPT7, and -SEPT11 polyclonal antibodies (Supplemental Table 4) with 1% BSA overnight at 4°C, washed with TBS, and incubated with Alexa Fluor 488–conjugated secondary antibody along with Hoechst, in a dark, humidified chamber for 2 hours at room temperature. Sections were washed again with TBS buffer, and slides were mounted and imaged using a Nikon Eclipse 90i confocal laser-scanning microscope. For background fluorescence controls, tissue sections were stained with Alexa Fluor 488–conjugated secondary antibody in the absence of primary antibodies.

For cryosections, mouse eyes derived from the control and h*SEPT9*-expressing groups after the completion of IOP recording (~5 weeks) were fixed in 4% buffered paraformaldehyde for 24 hours at 4°C, transferred into 5% and 30% sucrose in PBS on consecutive days, embedded in optimal cutting temperature (OCT) embedding media (Tissue-Tek), and cut into 10-μm-thick sections using a CM 1950 Cryostat (Leica Biosystems). Air-dried sections were treated with Image-iT FX signal enhancer (Invitrogen) and blocked using a blocking buffer (5% globulin-free BSA and 5% filtered goat serum in 0.3% Triton X-100–containing PBS) for 30 minutes. Tissue sections were then incubated overnight with the respective primary antibodies (Supplemental Table 4) at 4°C in blocking buffer. Sections were then washed in 0.3% Triton X-100–containing PBS prior to incubation with appropriate secondary antibodies conjugated with Alexa Fluor 488 or 594. For F-actin staining, as mentioned above, preblocked sections were incubated with phalloidin-TRITC for 2 hours at 1:500 dilution, washed, and mounted as described above. Additionally, the representative tissue sections from both control and h*SEPT9*-expressing groups were evaluated for the expression of inflammatory markers by immunostaining for Iba-1 (using rabbit polyclonal antibody, FUJIFILM Wako Chemicals), and F4/80 (BioLegend) conjugated with FITC, as described previously (15). All immunofluorescence data presented in this study are representative and based on the analysis of at least 4 tissue sections from 2 independent specimens. Images were captured as described above.

To quantify fluorescence in cryosections, consistent imaging conditions were applied to stained sections from both control LV and h*SEPT9* LV–expressing mouse eye anterior chambers. Images (1024 × 1024, 12-bit) of all *Z*-stacks were captured and imported into NIS-Elements AR5.42.01 software. Using the ROI drop-down menu, the Bezier ROI was selected to manually outline the TM and inner wall of the SC region. The ROI statistics function was then used to measure the area and mean fluorescence intensity of each pixel across all *Z*-stacks, along with the SD. Background fluorescence was determined from a blank region and subtracted from each measurement to ensure accuracy. The adjusted mean pixel intensity values were exported to a Microsoft Excel spreadsheet, and fold changes were calculated using GraphPad Prism. A minimum of 4 measurements were taken to determine the average pixel intensities for each protein. All fluorescence images shown are maximum intensity projections.

### Cell viability assay

To assess cell viability and cytotoxicity under the conditions treated with latrunculin A, Y27632, and UR214-9, live cell analyses were conducted using cell-permeable fluorescein diacetate and propidium iodide (343209 and 537059 respectively, MilliporeSigma), as previously described (5). Representative images showing merged green and red fluorescence were used to evaluate cell viability and toxicity.

### qRT-PCR

To assess the impact of *SEPT9* gene suppression on the expression of other septin subtypes, TM cells (2 strains, each in triplicate) were transfected with SEPT9 siRNA or scrambled siRNA as previously described (43). After 72 hours, cells were harvested for RNA extraction using the RNeasy Micro Kit (Qiagen). RNA was reverse transcribed with the Advantage RT for PCR Kit (Takara Bio USA). The resulting single-stranded cDNA libraries were used in PCR reactions with iQSYBR Green Supermix (Bio-Rad). Gene-specific primers for *SEPT9* (5′-GACCTGGGCGTGAAGAAC-3′ and 5′-GCTTGGGCATCTGGATCTC-3′), *SEPT11* (5′-CTCACACCCGCCACTATG-3′ and 5′-GGGCCTGGGACTGTAGTA-3′), *SEPT7* (5′-CAGACGTCAGATGCCTGATAAC-3′ and 5′-CCTACCACAGCAAGAGGTAAAC-3′), and *GAPDH* (5′-GGAGAAACCTGCCAAGTATGA-3’ and 5’-CCTCAGTGTAGCCCAAGATG-3′) were used in the reactions. PCR was conducted in triplicate using the Quant Studio 3 Real-Time PCR Detection System (Applied Biosystems/Thermo Fisher Scientific), as described previously (43). *SEPT9*, *SEPT11*, and *SEPT7* gene expression levels were normalized to *GAPDH* and analyzed using the comparative threshold method.

### Cell contraction assay

The collagen-based gel contraction assay was performed using the Collagen-Based Gel Contraction Assay Kit (CBA-201, Cell Biolabs Inc.). TM cells (2 strains and their multiple replicates) were infected with LVs expressing either *eGFP* alone (control LV) or h*SEPT9* and *eGFP* (LV-hSEPT9), as previously described. After 1 week of infection, the cells were harvested and mixed with collagen lattice solution at a 1:4 ratio according to the manufacturer’s instructions. This mixture was then plated into a 24-well plastic plate and incubated at 37°C for 1 hour to allow collagen polymerization. Following polymerization, 1 mL of growth media was added to each well, and the plates were incubated for an additional 48 hours at 37°C. To initiate contraction, the gels were detached from the sides of the culture dish and imaged using the ChemiDoc Touch imager. The diameter (*D*) of the gels was manually measured from the captured images, and the circumference was calculated using the formula *C* = π*D*.

### Immunoprecipitation and mass spectrometry

To identify SEPT9-interacting proteins in human TM cells, immunoprecipitation analyses were performed using an anti-SEPT9 polyclonal antibody (Supplemental Table 1) with Dynabeads Protein A (10001D, Thermo Fisher Scientific) along with the respective IgG control, as per the manufacturer’s instructions described previously (62). Immunoprecipitates were solubilized in 2% SDS, 100 mM Tris-HCl (pH 8.0), reduced with 10 mM DTT, alkylated with 25 mM iodoacetamide, and subjected to tryptic hydrolysis using the HILIC beads SP3 protocol (ReSyn Biosciences), as previously described (62). The protein digests were separated by liquid chromatography using the Q Exactive HF Orbitrap mass spectrometer (Thermo Fisher Scientific), as previously described (17). For protein identification and label-free relative protein quantification, raw mass spectral data files were imported into Progenesis QI for Proteomics 4.2 software (Nonlinear Dynamics) for duplicate run alignment of each preparation and peak area calculations, as previously described (17). Only proteins identified on the basis of 2 or more peptides (protein confidence *P* < 0.05 and false discovery rate < 1%) were included in the protein quantification analysis.

### Phagocytosis analysis

To determine the impact of *SEPT9* overexpression on phagocytosis, TM cells were infected with the LVs mentioned above for 8 days. The culture medium was subsequently replaced with complete media supplemented with pHrodo Red *E*. *coli* BioParticles Conjugate (P35361, Thermo Fisher Scientific) at a final concentration of 0.1 mg/mL. After incubating the cells at 37°C in the cell culture incubator for 2 hours, coverslips were transferred to phenol red–free media, and images were captured to detect red fluorescence using a Nikon Eclipse Ts2R microscope.

For flow cytometry–based quantification analysis, 2 separate sets of primary cultures were similarly treated with LVs. At the end of the infection period as mentioned above, cells were incubated with pHrodo Green *E*. *coli* BioParticles Conjugate. Subsequently, cells were trypsinized, transferred to sterile Eppendorf tubes, and centrifuged at 1000*g* for 10 minutes. The cell pellets were then washed and suspended in 1× PBS, and loaded onto an Aurora flow cytometer (Cytek Biosciences) for data collection. Each sample consisted of 100,000 cells, and appropriate controls were included, such as cells expressing neither eGFP nor pHrodo Green, cells expressing eGFP alone, and LV-control with pHrodo Green. The data were stored as an FCS file, unmixed, and analyzed using SpectroFlo software (Cytek Biosciences).

### Cell culture permeability analysis

To assess whether the septin cytoskeleton influences TM cell permeability, TM cells were plated on tissue culture–treated Transwells (12 mm Transwell inserts with 0.4 μm pore polyester membrane, 3460, Costar) and allowed to grow in 10% FBS–containing complete media as described above. Cells were allowed to reach complete confluence and continued to grow for an additional 5 days, after which serum was dropped to 1% and maintained for 2 hours as mentioned above, prior to treatment with 10 μM UR214-9 for 18 hours. For a positive control, cells plated and maintained similarly were treated with 10 μM Rho kinase inhibitor Y27632 for 2 hours. Dimethyl sulfoxide–treated (vehicle-treated) and untreated cultures maintained under the same conditions were used as controls for UR214-9 and Y27632, respectively. Following this treatment, the Transwell inserts were transferred to a new multiwell plate and 0.8 mL of phenol red–free minimum essential medium (MEM; 51200-038, Gibco/Invitrogen) was added to the basolateral compartment and the apical compartment media were replaced with MEM containing 250 μL FITC-dextran suspension (1 mg/mL; FD20S, MilliporeSigma), and the Transwells were moved back into the cell culture incubator for 20 minutes. At the end of the incubation period, 0.1 mL of medium (in triplicate) from the basolateral chamber was collected into 96-well clear bottom microplates (3603, Corning). Similarly, various concentrations of FITC-dextran solution aliquoted (0.1 to 50 μg/mL) into the 96-well plates served as a standard and the fluorescence intensity was measured at 490/520 nm excitation/emission using a SpectraMax M3 plate reader (Molecular Devices). The relative FITC-dextran fluorescence per unit of medium determined for the test and control samples was analyzed. Following the completion of the permeability assays, the cells on the Transwell filters were fixed, permeabilized, and stained for F-actin using phalloidin-TRITC and imaged as described above.

### Mice and IOP recording

Studies were conducted using male and female adult wild-type mice of the C57BL/6J strain, aged 4 months old. The mice were provided ad libitum access to food and water and were housed in an environment maintained at 21°C with a 12-hour light/dark cycle.

Viral vectors were injected bilaterally into either control or test groups, each consisting of 6 mice. The LVs expressing *eGFP* alone (control LV) or those expressing human *SEPT9* and *eGFP* (LV-h*SEPT9*) under a CMV promoter (custom made by VectorBuilder Inc) were injected under ketamine/xylazine anesthesia at a concentration of 3.6 × 10^8^ transduction units/mL in 3 μL. After recording the baseline IOP, LV injections were performed, and IOP measurements were carried out diurnally (between 1 and 3 pm) using a Tonolab rebound tonometer (iCare Laboratory) under mild isoflurane anesthesia. IOP was recorded twice per week, as previously described (15). LV injections were repeated after 2 weeks in the same mice, and IOP monitoring was continued for an additional 2.5 weeks. At the end of IOP recording, the mice were euthanized, and enucleated eyes were fixed for either cryosections or tissue histology (15). IOP changes were compared between the control and test groups at indicated time points.

### Tissue histology

Enucleated eyes derived from the control LV– and LV-h*SEPT9*–injected groups after the completion of IOP recording (~5 weeks) were fixed in 2% paraformaldehyde and 2% glutaraldehyde in PBS for 24 hours at 4°C. After removing the fixative, eyes were washed with PBS and dissected. The anterior segments of the eyes were divided into quadrants and processed following a standard TEM protocol (15). The tissue specimens were cut into 0.5-μm sections using a Reichert Ultra-cut microtome (Leica), stained with 1% methylene blue, visualized under light microscopy, and images captured with a Zeiss Axioplan2 microscope. The ultra-thin sections (65–75 nm) obtained using a Leica EM UC7 ultramicrotome on grids were contrast stained with 2% uranyl acetate and 3.5% lead citrate solution. The sections were visualized, and images captured at ×8000 magnification using a Jeol JEM-1400 electron microscope equipped with Orius CCD digital camera at 60 kV.

To evaluate changes in the SC lumen area, we conducted a morphometric analysis using the Bezier ROI tool as described above. Additionally, we manually counted both large and small vacuoles in the inner wall of the SC from TEM micrographs of mouse eyes, both from control and h*SEPT9*-expressing groups. For each group, we analyzed a minimum of 2 quadrants per eye, with a total of 4 mice per group.

### Association of the SEPT9 genetic variant with IOP in GERA cohort

The GERA cohort consists of 110,266 adults who consented to participate in the Research Program on Genes, Environment, and Health, established for members of the Kaiser Permanente Medical Care Plan, Northern California Region (KPNC). We tested the lead genetic variant rs9038 at *SEPT9* identified in a previous GWAS of IOP (18) for replication in the GERA non-Hispanic White sample, consisting of 56,819 individuals with IOP measurements (63). GWAS summary statistics for IOP from the study of Choquet et al. (63, 64) were used for the association analysis. The regional plot at the *SEPT9* locus was generated using the LocusZoom web-based plotting tool (65, 66).

### Statistics

Statistical analyses were performed using Prism software (version 9.3.1; GraphPad Software). Comparisons between 2 groups were conducted using a 1-tailed Student’s *t* test. For cell culture–based experiments, quantitative results are expressed as the mean ± SEM, with data from the experimental group reported as fold change relative to the control group. Control group results were normalized to a fold change of 1. The nonparametric Wilcoxon’s rank-sum test was used to compare IOP changes between mice injected with LV-h*SEPT9* and those injected with control LV. A *P* value of less than 0.05 was considered statistically significant for all analyses.

### Study approval

#### Mice.

All mouse experiments conducted in this study adhered to the recommendations of the NIH *Guide for the Care and Use of Laboratory Animals* (National Academies Press, 2011). The animal protocol (A213-19-10) received approval from the Institutional Animal Care and Use Committee of the Duke University School of Medicine.

#### Human eye tissue.

The use of human tissue obtained from the donor eyes was approved by the Institutional Review Board of Duke University School of Medicine (Pro00050810) in compliance with Health Insurance Portability and Accountability Act guidelines, and the tenets of the Declaration of Helsinki.

#### Human participants.

The Research Program on Genes, Environment, and Health (GERA), established for members of the KPNC, and the Institutional Review Board of the Kaiser Foundation Research Institute have approved all study procedures.

### Data availability

All described data are included in the manuscript and in the supplemental material. Additionally, details of this study can be made available by the corresponding authors upon request. The complete GERA data are available upon application to the KP Research Bank (https://researchbank.kaiserpermanente.org/). Values for all data points in graphs are reported in the Supporting Data Values file.

## Author contributions

RM, KSN, HC, and PVR designed the research studies. RM, PG, LKL, PC, and NPS conducted experiments. RM, PG, LKL, NPS, HC, and KSN acquired data. RM, PG, PC, NPS, KSN, HC, and PVR analyzed data. RKS provided reagents, including UR214-9. RM, HC, and PVR wrote the manuscript. RM, HC, and PVR obtained funding. All authors edited the manuscript.

## Supplementary Material

Supplemental data

Unedited blot and gel images

Supplemental tables 2 and 3

Supporting data values

## Version 1 12/06/2024

Electronic publication
