# Supplementary material for: Dysregulation of septin cytoskeletal organization in the trabecular meshwork contributes to ocular hypertension
Source: JCI Insight. 2024 Dec 6;9(23):e179468. doi: 10.1172/jci.insight.179468 (PMC11623952; doi:10.1172/jci.insight.179468)
Supplement: Supplemental data [file jciinsight-9-179468-s106.pdf]

Dysregulation of septin cytoskeletal organization in the trabecular meshwork contributes to ocular hypertension

Rupalatha Maddala,[1*] Pallavi Gorijavolu,[1] Levi Lankford,[1] Nikolai P. Skiba,[1] Pratap Challa,[1] Rakesh K. Singh,[2] K. Saidas. Nair,[3] Hélène Choquet,[4] and Ponugoti V. Rao.[1, 5] *

Supplemental material

- **Supplemental Figures 11**
- **Supplemental Tables 2**

Supplemental Figure 1.

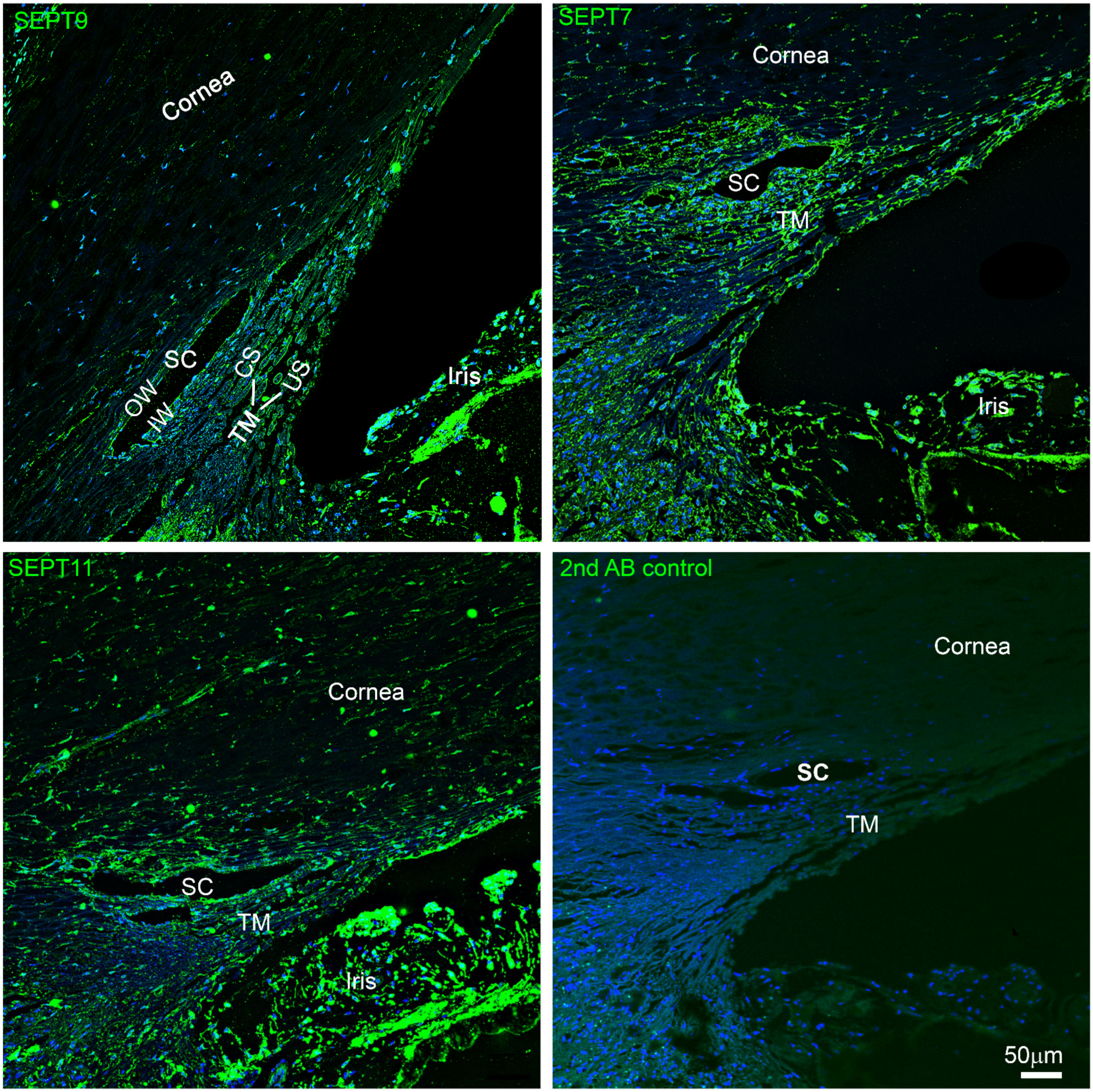

Supplemental Figure 1. **Distribution of SEPT9, SEPT11, and SEPT7 in the trabecular outflow pathway of the human eye, as determined by immunofluorescence analysis.** Primary antibodies were used in conjunction with a secondary antibody conjugated to a 488 fluorophore (green). The bottom right panel displays non-specific background fluorescence from a tissue section stained with the secondary antibody alone. Blue staining represents cell nuclei labeled with Hoechst. Abbreviations: TM, trabecular meshwork; SC, Schlemm's canal. US: Uveoscleral, CS: Corneoscleral, IW: Inner wall, OW: Outer wall. Scale bar indicates image magnification.

Supplemental Figure 2.

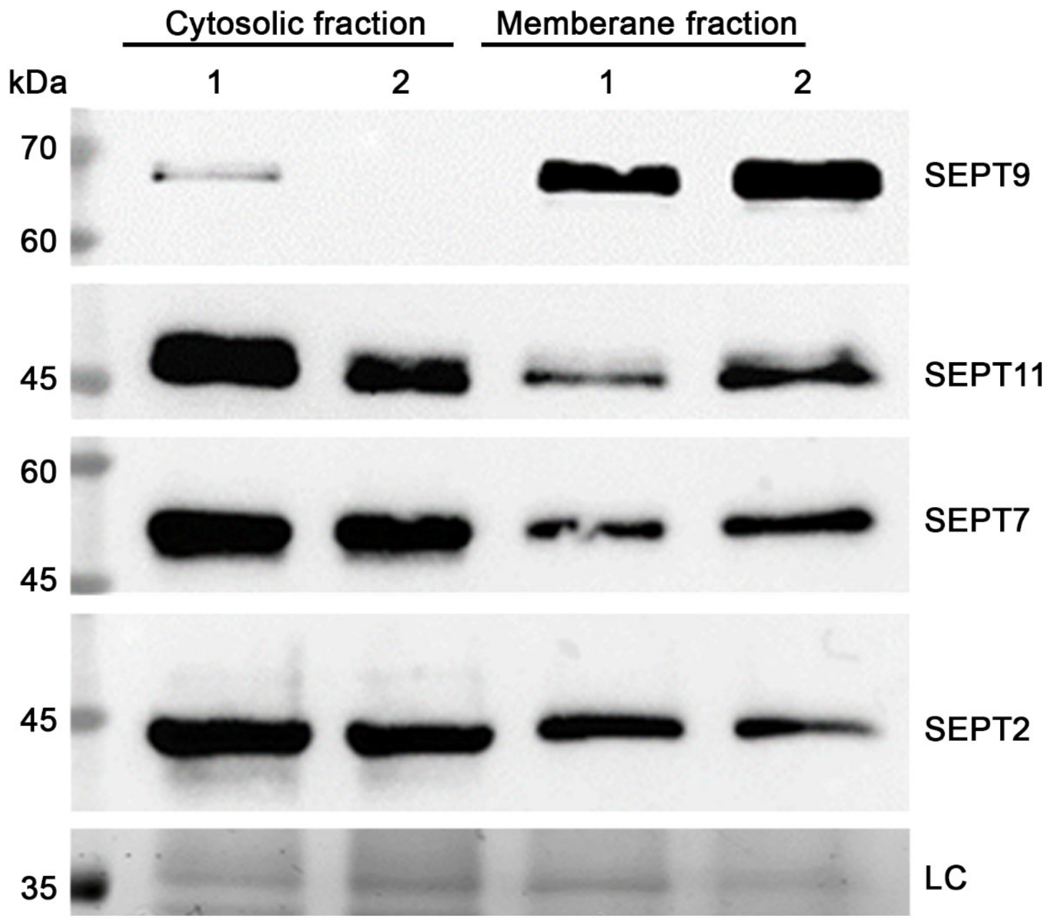

Supplemental Figure 2. Distribution of septins in the cytosolic and membrane-enriched fractions of human TM cells. LC: loading control. Equal amounts of protein from the indicated fractions were separated on an SDS-PAGE gel, stained with Gel code blue, and one of the stained bands was used as the loading control.

Supplemental Figure 3.

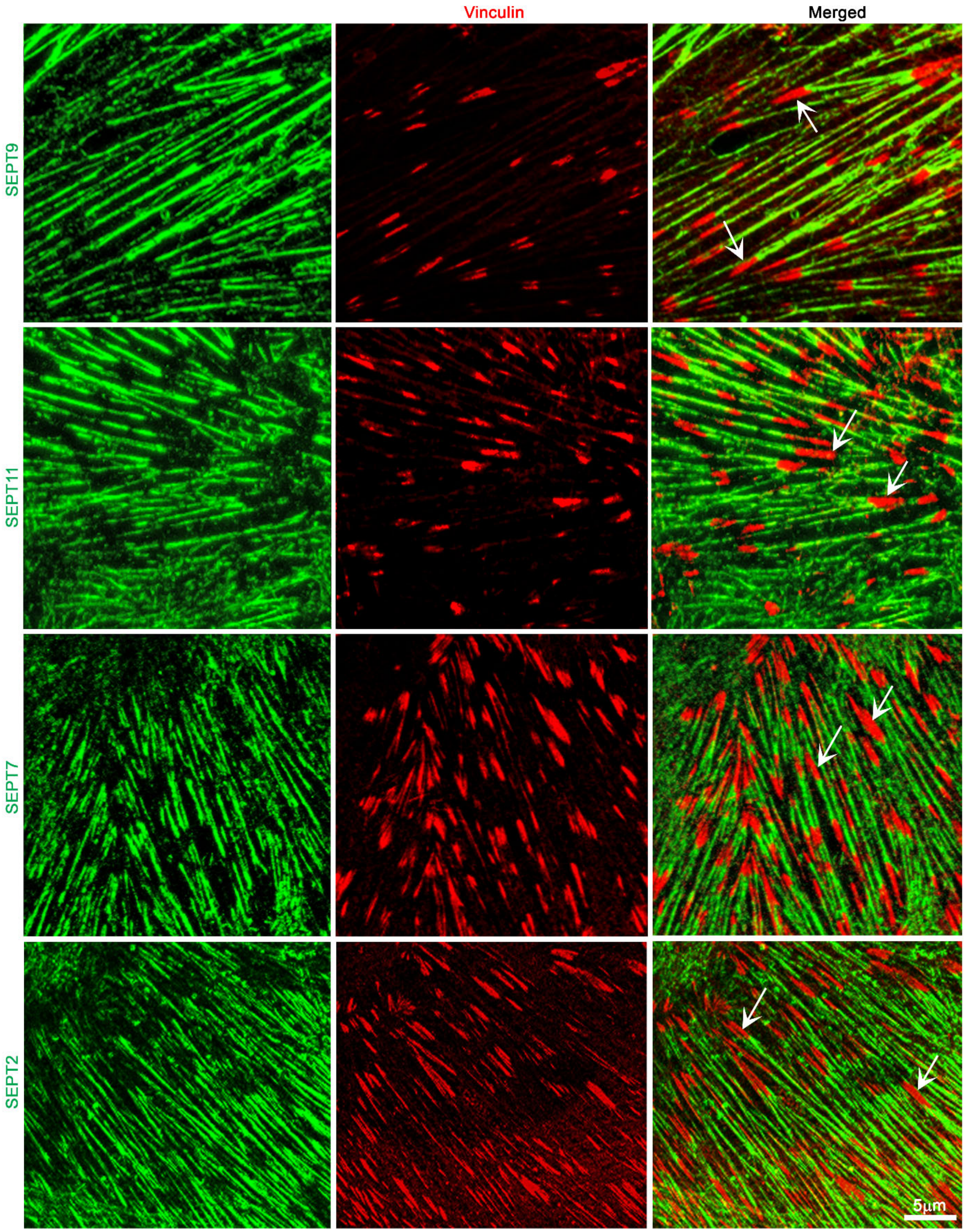

Supplemental Figure 3. Distribution of SEPT9, SEPT11, SEPT7, SEPT2, and vinculin in human TM cells. To investigate whether vinculin (a focal adhesion protein) colocalizes with SEPT9, SEPT11, SEPT7, and SEPT2 in TM cells, we performed colocalization analysis using immunofluorescence imaging. Arrows in the merged images indicate no obvious colocalization between vinculin and septins. Scale bar indicates image magnification.

Supplemental Figure 4.

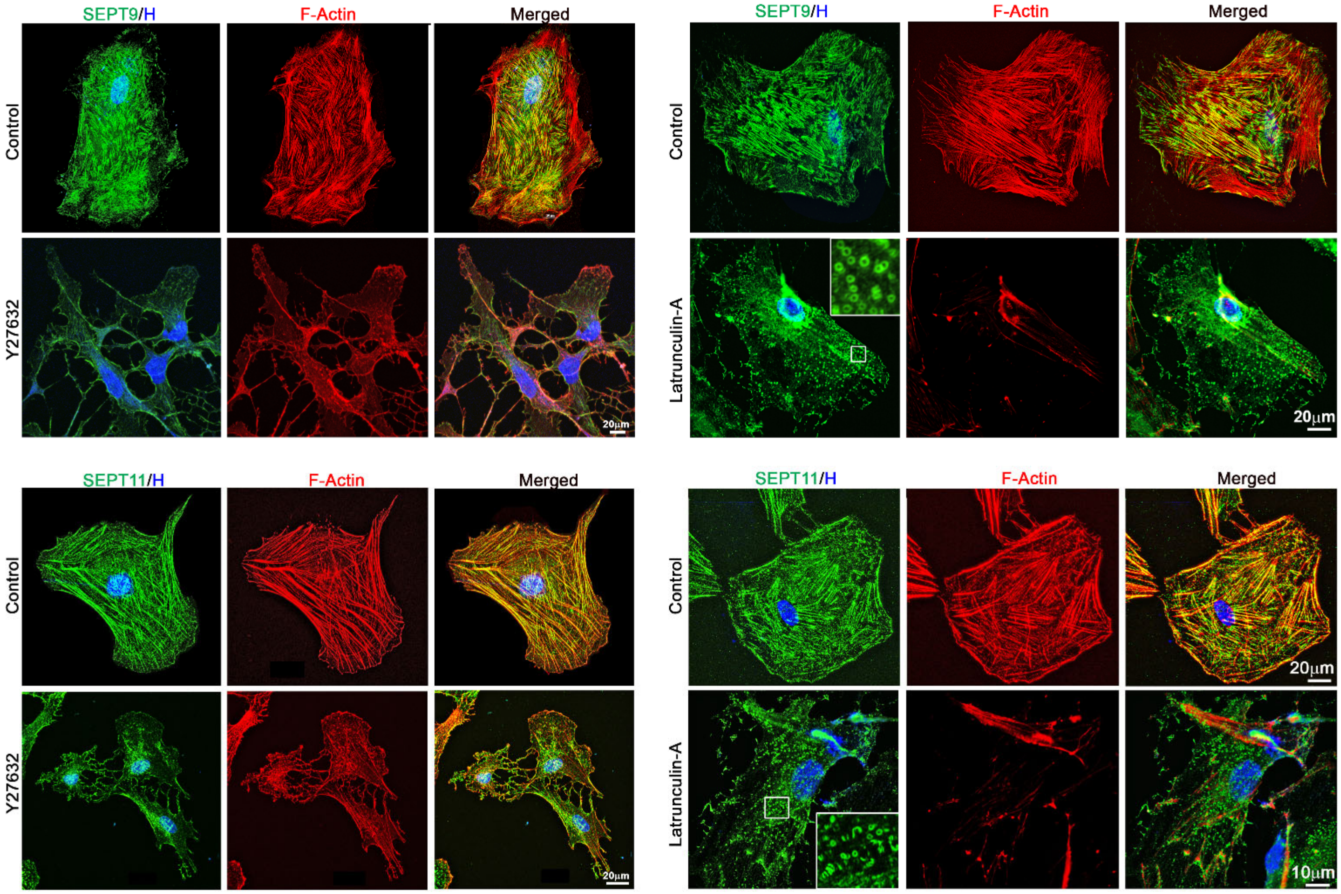

Supplemental Figure 4: Actin depolymerization disrupts SEPT9 and SEPT11 filamentous organization in human trabecular meshwork (TM) cells treated with the Rho kinase inhibitor Y27632 and Latrunculin-A. Cell treatments were performed as described in the Methods section. SEPT9 and SEPT11 were immunostained with the polyclonal antibodies, F-actin was labeled with phalloidin-TRITC, and cell nuclei were stained with Hoechst (blue). Both Rho kinase inhibitor and Latrunculin-A lead to a reduction and disruption of SEPT9 and SEPT11 filament organization in TM cells, which is associated with a depolymerized actin cytoskeleton. Latrunculin-A treatment induces a ring-like SEPT9 organization, which is shown in magnified images of the boxed area (see inserts). Scale bar indicates image magnification.

Supplemental Figure 5.

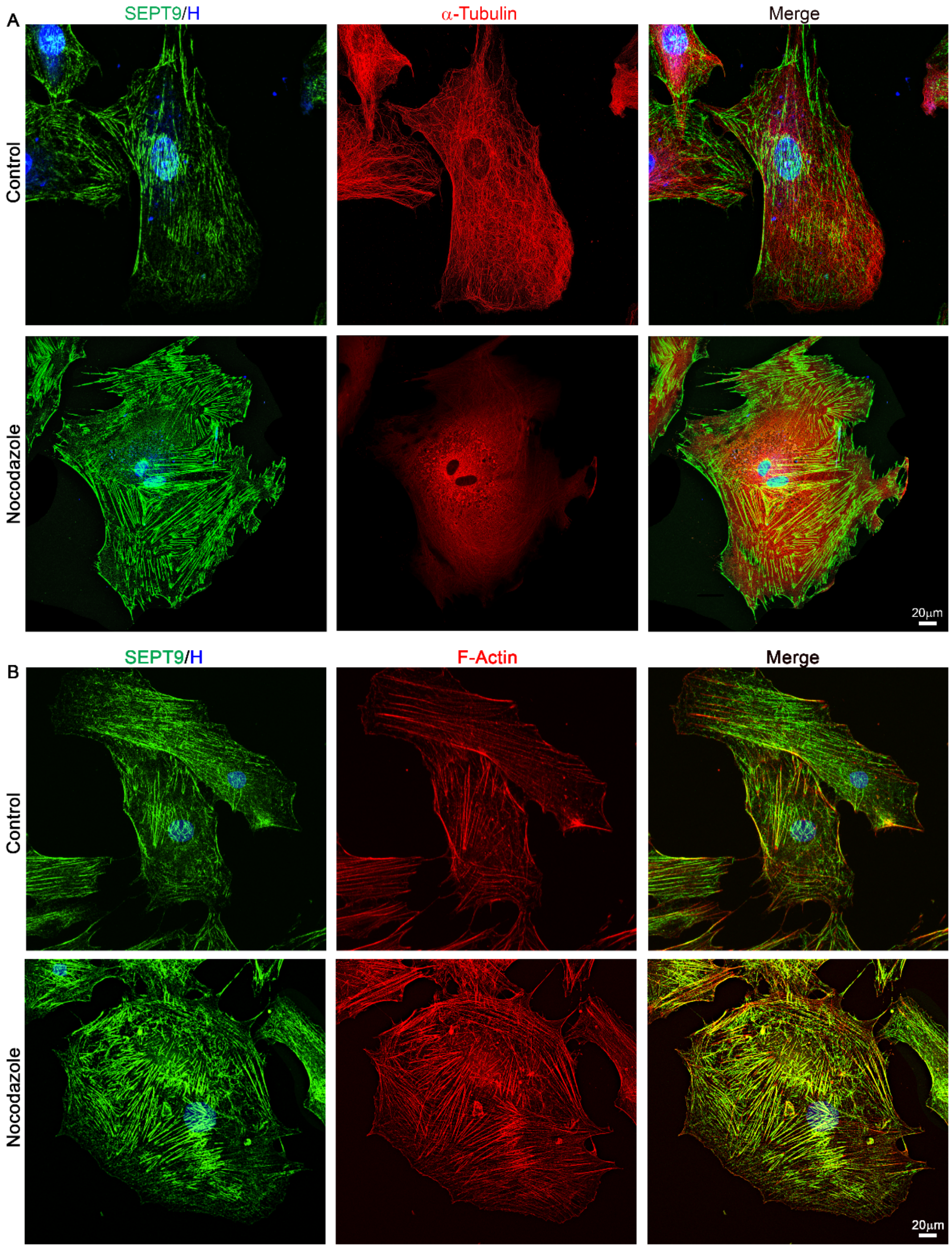

Supplemental Figure 5. Colocalization of SEPT9 with microtubules and the effect of microtubule depolymerization on SEPT9 filament formation in human TM cells. A. TM cells were co-immunostained for SEPT9 and α-tubulin using specific antibodies. To examine how microtubule depolymerization affects SEPT9 filament organization, cells were treated with nocodazole (5 μM for one hour) and immunostained for changes in microtubule organization and SEPT9 distribution. The results show that SEPT9 colocalizes with microtubules, and microtubule depolymerization induces the formation of SEPT9 filaments.

B. Microtubule depolymerization also leads to the formation of actin stress fibers, as indicated by phalloidin-TRITC staining. These actin stress fibers colocalize with SEPT9 filaments. Cell nuclei are stained with Hoechst. Scale bar indicate image magnification.

Supplemental Figure 6.

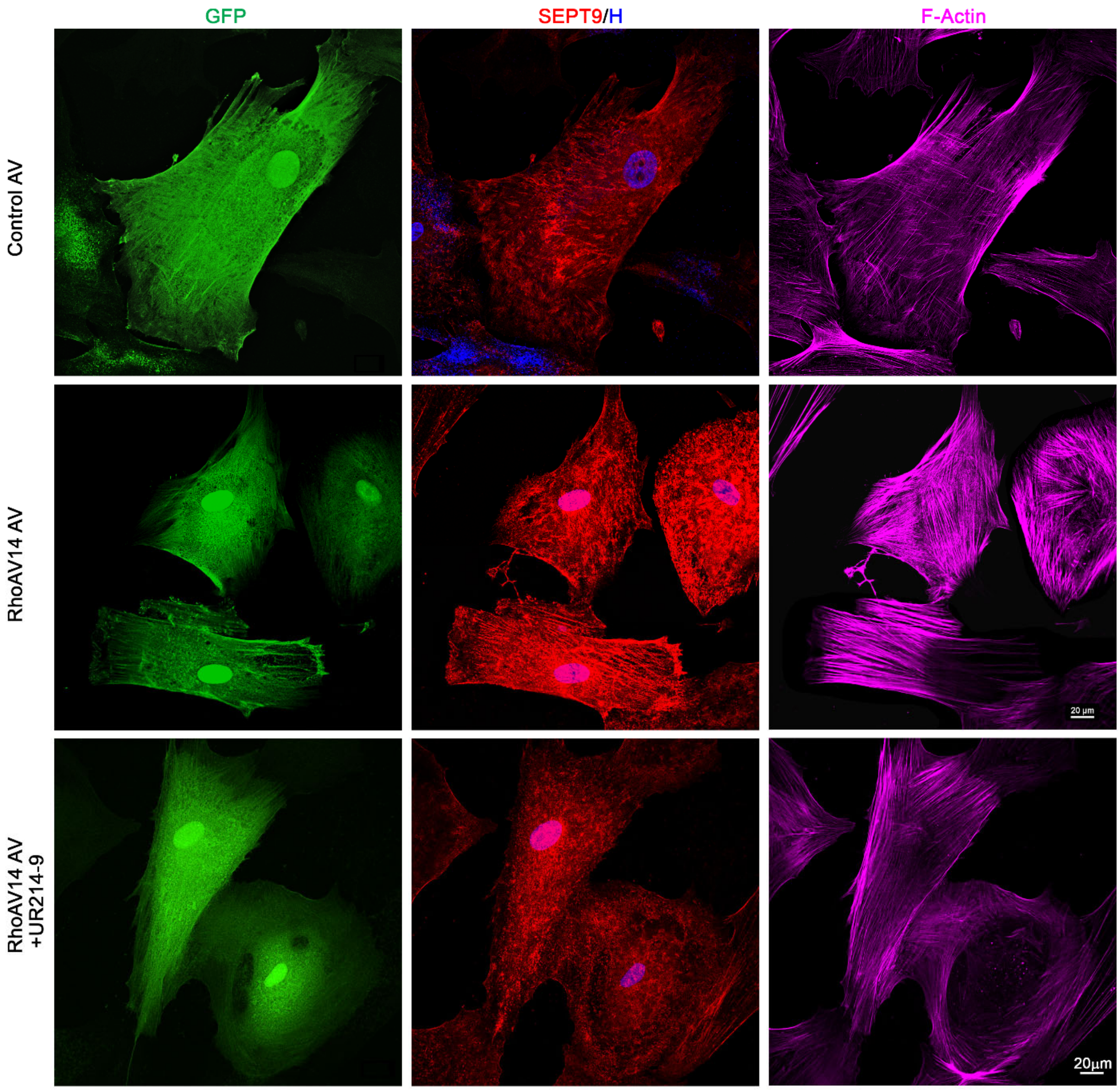

Supplemental Figure 6. Constitutively active RhoA induces SEPT9 filament formation, which is disrupted by UR214-9 in TM cells. TM cells expressing constitutively active RhoA induces actin stress fibers and SEPT9 filaments, as detected by far-red (Alexa Fluor™ 647)-phalloidin fluorescence and immunofluorescence, respectively. However, the presence of the septin cytoskeletal disruptor UR214-9 (10 µM for 24 hours) suppresses RhoA-induced SEPT9 filament formation and decreases actin stress fibers. Cell nuclei are stained with Hoechst. Scale bars indicate image magnification.

Supplemental Figure 7.

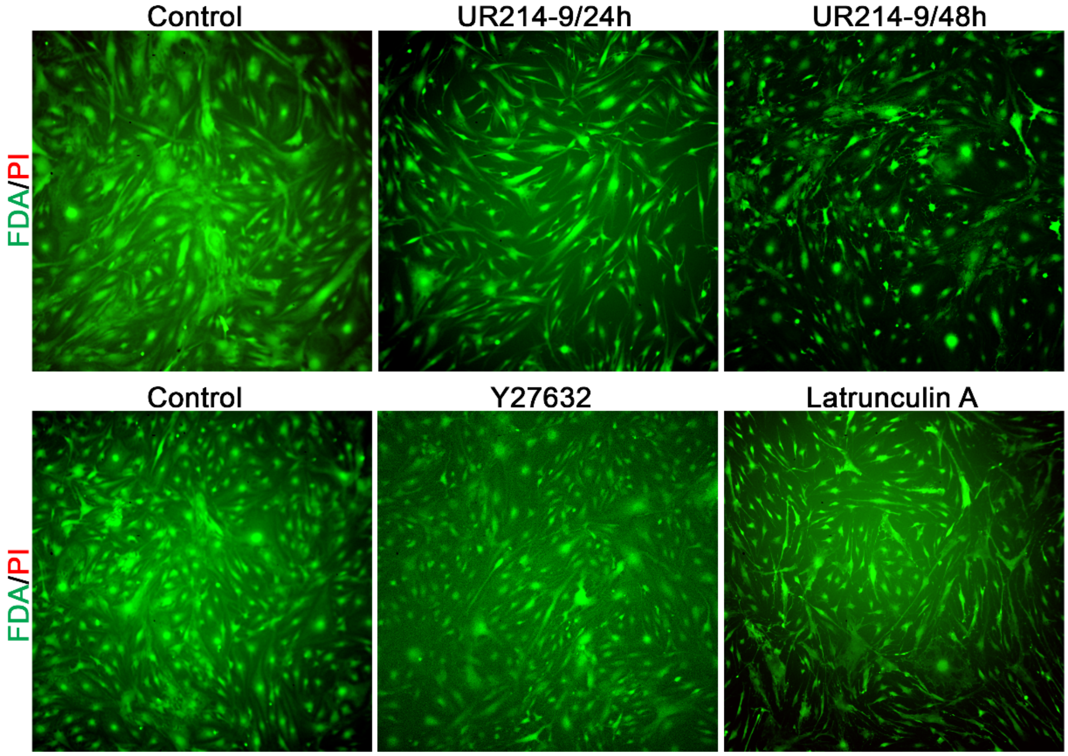

Supplemental Figure 7. Cell viability and toxicity analysis in TM cells treated with UR214-9, Y27632, and Latrunculin-A. Cell viability was assessed using live cell imaging with fluorescein diacetate (FDA) and propidium iodide (PI). FDA, which permeates live cells, produces green fluorescence, indicating cell viability. PI, which stains only dead cells, produces red fluorescence in the nuclei of non-viable cells. As detailed in the Methods section, imaging of both control and drug-treated cells revealed no evidence of cell toxicity, as indicated by the absence of red PI staining and the presence of green fluorescence from FDA, confirming cell viability.

Supplemental Figure 8.

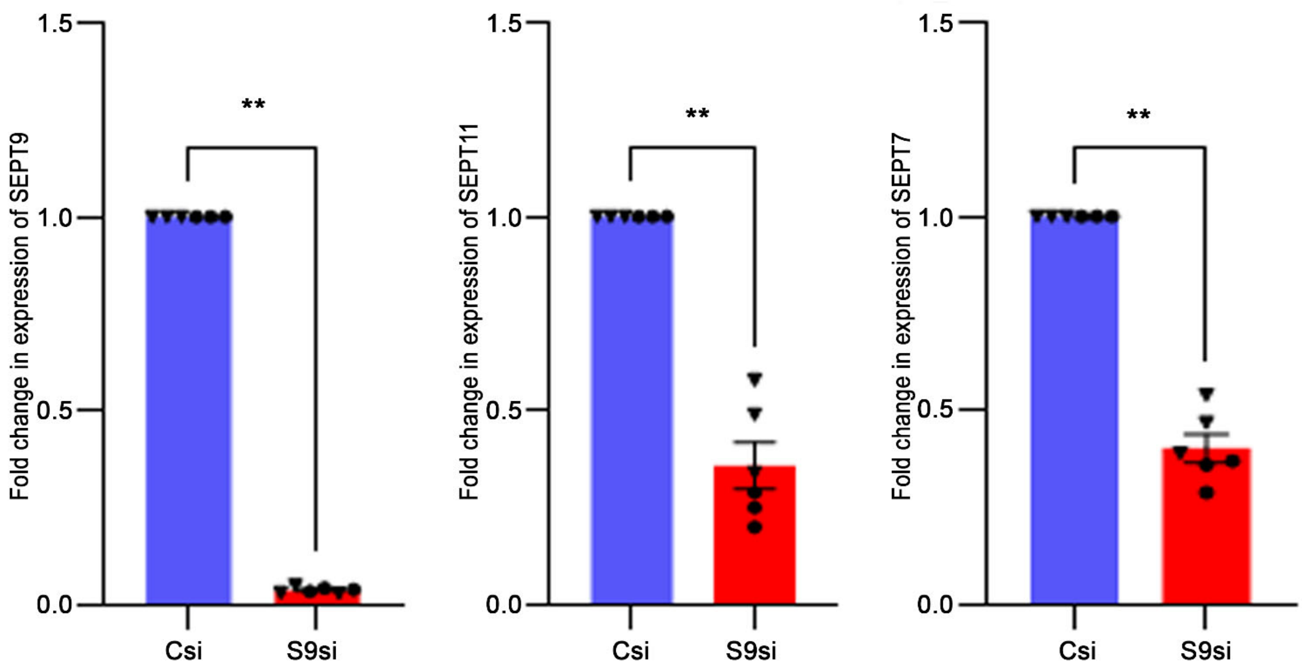

Supplemental Figure 8. Suppression of *SEPT9* expression reduces the levels of other septins in TM cells.

Immunoblot analyses (shown in Figure 4) revealed that reduced *SEPT9* expression leads to decreased SEPT11 and SEPT7 levels in TM cells. To further investigate this, qRT-PCR analysis was conducted to assess the effects of *SEPT9* knockdown on *SEPT11* and *SEPT7* expression in TM cells. RNA was extracted from TM cells transfected with SEPT9 siRNA, as detailed in the Methods section. qRT-PCR results showed that SEPT9 siRNA transfection significantly reduced *SEPT9* expression by 80% and decreased *SEPT11* and *SEPT7* levels by over 50% compared to control cells transfected with scrambled siRNA. Error bars represent the mean ± SEM of six samples (triplicates from two different strains). **P < 0.001, Student's t-test.

Supplemental Figure 9.

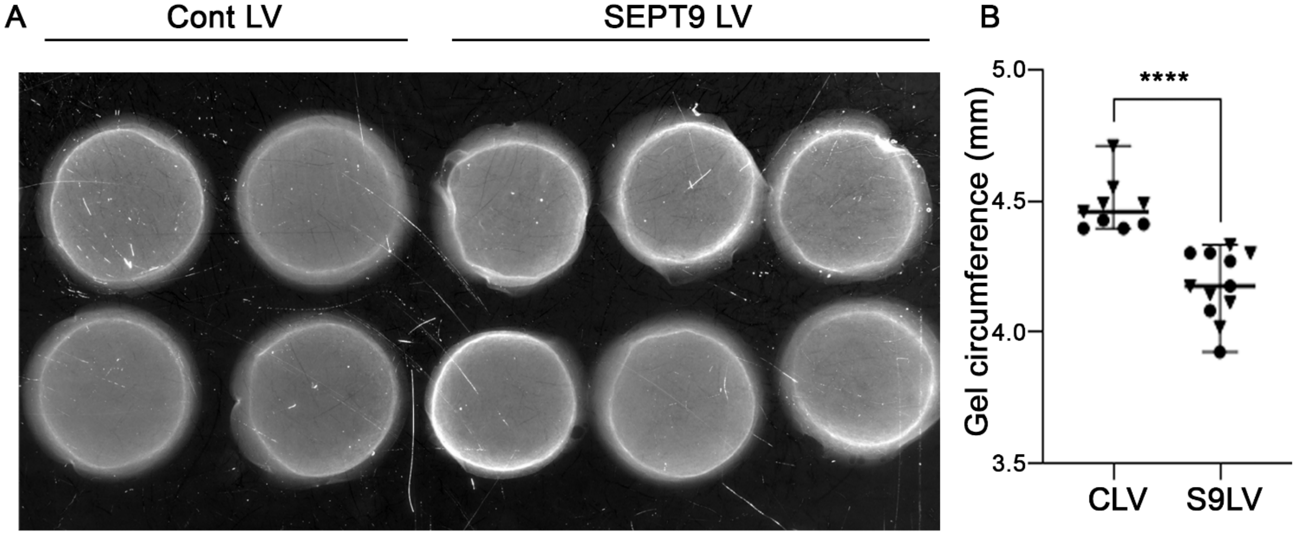

Supplemental Figure 9. Increased expression of *SEPT9* in TM cells induces collagen-gel contraction.

Altered expression of SEPT9 in TM cells significantly affects actin stress fiber formation, focal adhesion activation, and myosin light chain phosphorylation, indicating SEPT9's role in modulating contractile characteristics. To further explore this, a collagen gel contraction assay was performed as described in the Methods section, using TM cells expressing recombinant SEPT9, compared to GFP-expressing control cells.

A & B. TM cells with increased *SEPT9* expression (*SEPT9* LV infected; S9LV) exhibited significantly greater gel contraction (evidenced by decreased gel circumference) compared to *eGFP*-expressing control cells (control LV infected; CLV). Panel A displays representative images of collagen gels. Error bars represent mean ± SEM for N=8-10 (two strains). **** P<0.0001, based on a Student’s t-test.

Supplemental Figure 10.

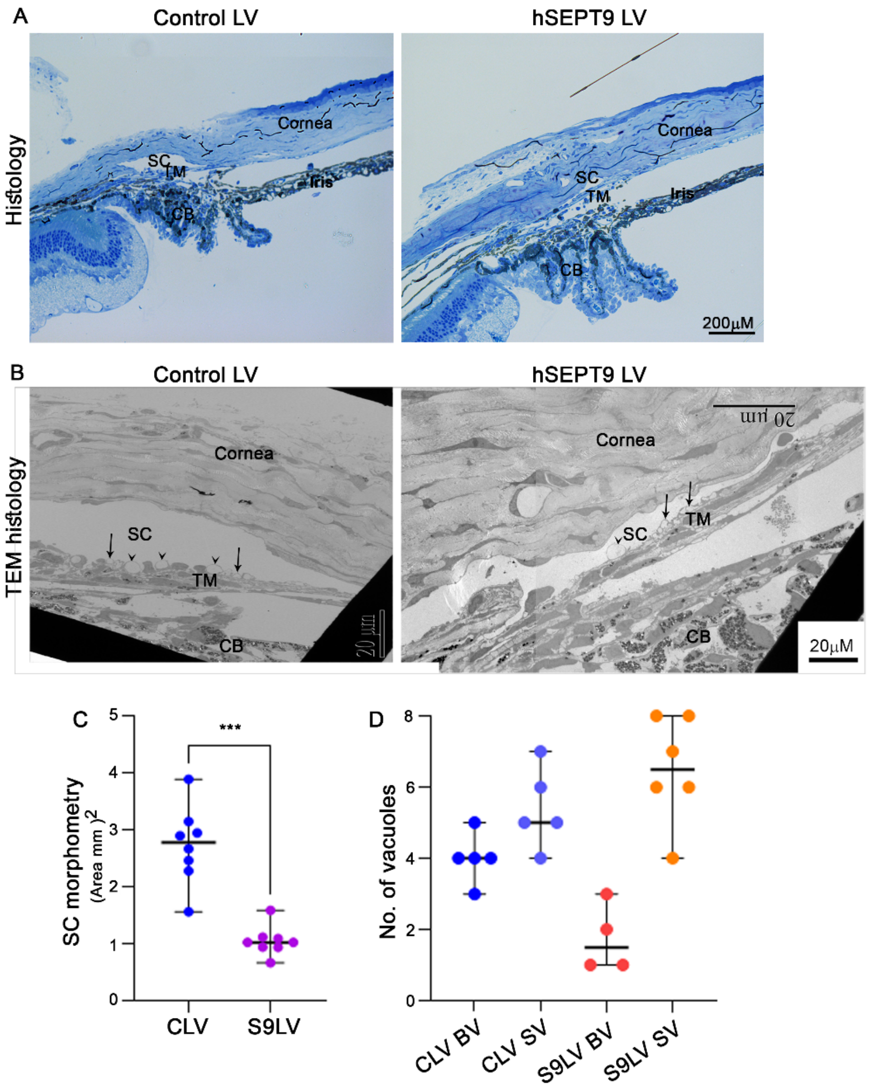

Supplemental Figure 10. **Impact of Increased *SEPT9* Expression on the Geometry of the Trabecular Outflow Pathway in Mice.** Transmission electron microscopy (TEM) and subsequent morphometric analysis revealed a significant decrease in Schlemm's canal (SC) lumen area in h*SEPT9*-expressing specimens compared to *eGFP*-expressing controls.

A. Light microscopy images of histological sections from control (*eGFP*-expressing) and h*SEPT9*-expressing specimens.

B. TEM images of the trabecular pathway, including the trabecular meshwork (TM) and SC, in control and h*SEPT9*-expressing specimens.

C. Morphometric analysis quantifying the SC area in both control and h*SEPT9*-expressing specimens. In addition to the reduced SC lumen area, h*SEPT9*-expressing specimens exhibited firmer TM tissue compared to controls. There was also a noticeable decrease in the number of large vacuoles (BV, indicated by arrowheads) and an increase in the number of small vacuoles (SV, indicated by arrows) in h*SEPT9*-expressing specimens, as shown in panel B.

D. Displays the results of manual counts of BV and SV. Error bars represent the mean ± SEM of a sample size of 4 independent mice. ***$P < 0.001$, based on Student's t-test. Bar scale bar indicate image magnification. CLV, Control Lentiviral Vector; S9LV, SEPT9 Lentiviral Vector.

Supplemental Figure 11.

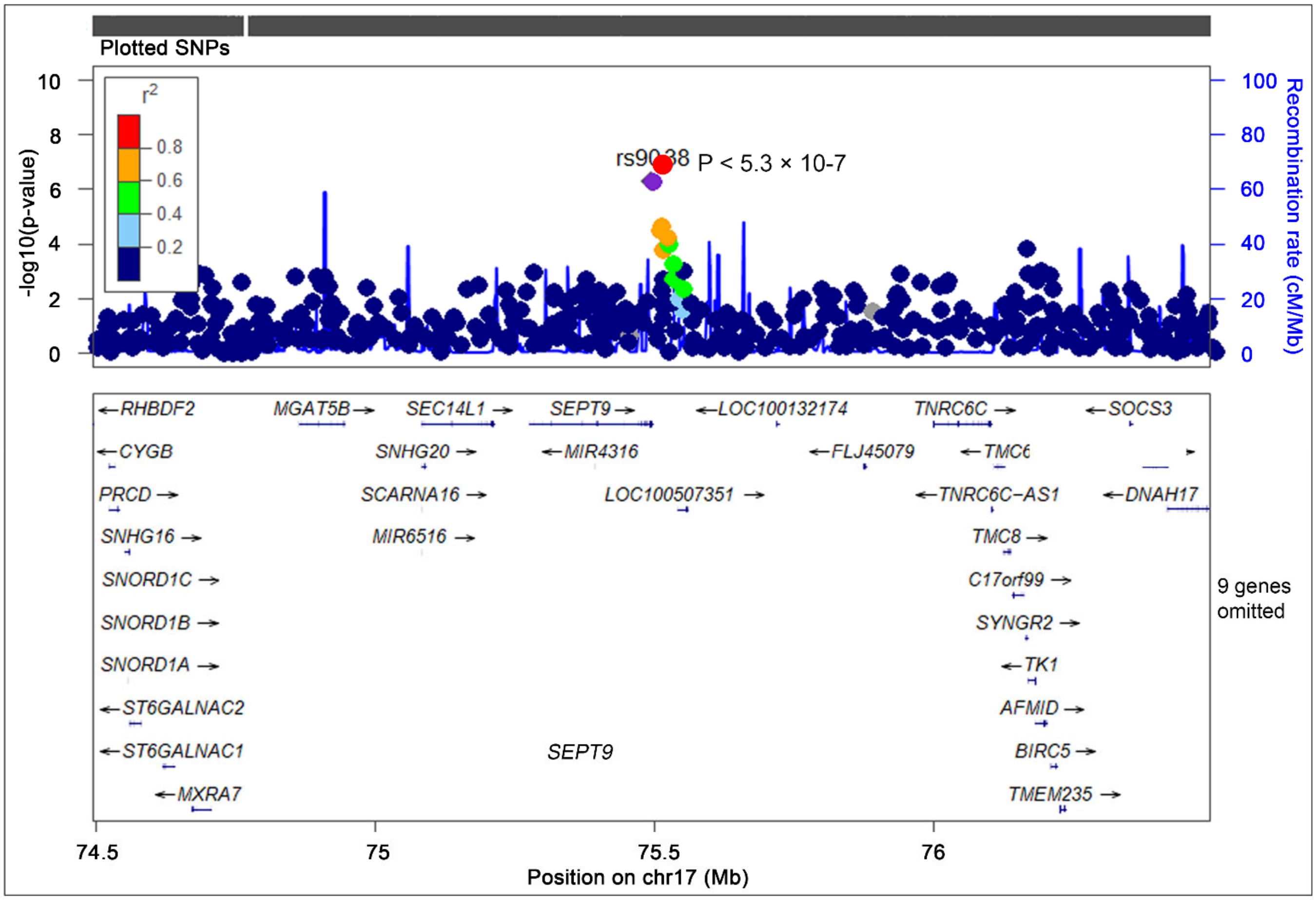

Supplemental Figure 11. Regional association plot for the *SEPT9* gene's association with IOP. (A) The *SEPT9* genetic variant rs9038, highlighted in red, shows a significant association with IOP in the GERA cohort.

Squares represent genotyped variants, while circles represent imputed variants. The genes are displayed below the SNPs (single nucleotide polymorphisms), with arrows indicating the strand orientation of each gene. The color-coding indicates the level of linkage disequilibrium with the lead SNP.

Supplemental Table 1: Identification of septin-9 co-immunoprecipitated proteins in human trabecular meshwork cells using Mass spectrometry

Protein	Gene	Ratio Sep9/IgG
Septin-9	*SEPT9*	23.25
Phostensin	*PPP1R18*	20.79
Septin-10	*SEPT10*	18.39
Unconventional myosin-Ib	*MYO1B*	15.01
Septin-11	*SEPT11*	11.9
Cytosolic phospholipase A2	*PLA2G4A*	10.76
Nexilin	*NEXN*	8.07
Fibrillin-1	*FBN1*	7.94
Cytoplasmic FMR1-interacting protein 1	*CYFIP1*	7.88
Septin-2	*SEPT2*	7.56
Septin-7	*SEPT7*	7.15
Fibronectin type-III domain-containing protein 3A	*FNDC3A*	7.09
Microtubule-actin cross-linking factor 1	*MACF1*	6.65
Dynamin-1-like protein	*DNM1L*	6.65
Drebrin	*DBN1*	6.19
Spartin	*SPART*	6.16
ATP-binding cassette sub-family F member 1	*ABCF1*	5.99
Dynamin-2	*DNM2*	5.95
AP-1 complex subunit gamma-1	*AP1G1*	5.9
Catenin delta-1	*CTNND1*	5.88
Cytoskeleton-associated protein 5	*CKAP5*	5.69
Alpha-adducin	*ADD1*	5.33
Unconventional myosin-Ic	*MYO1C*	5.26
Thrombospondin-1	*THBS1*	5.21
Neural cell adhesion molecule 1	*NCAM1*	5.12
Inverted formin-2	*INF2*	5.07
Catenin beta-1	*CTNNB1*	5.04
Exportin-1	*XPO1*	5.03
Dynamin-like 120 kDa protein, mitochondrial	*OPA1*	4.62
CD166 antigen	*ALCAM*	4.59
Cullin-associated NEDD8-dissociated protein 1	*CAND1*	4.38
Tensin-1	*TNS1*	4.36
Contactin-1	*CNTN1*	4.35
Band 4.1-like protein 2	*EPB41L2*	4.32
CCN family member 2	*CCN2*	4.31
Calcium/calmodulin-dependent protein kinase type II subunit delta	*CAMK2D*	4.26
Coatomer subunit gamma-1	*COPG1*	4.21
Myosin phosphatase Rho-interacting protein	*MPRIP*	4.12
Latent-transforming growth factor beta-binding protein 2	*LTBP2*	4.11
Cytoplasmic dynein 1 heavy chain 1	*DYNC1H1*	4.08

Moesin	*MSN*	4.00
AP-2 complex subunit alpha-1	*AP2A1*	3.97
CD59 glycoprotein	*CD59*	3.92
AP-2 complex subunit beta	*AP2B1*	3.86
LIM domain and actin-binding protein 1	*LIMA1*	3.81
Integrin beta-5	*ITGB5*	3.73
14-3-3 protein beta/alpha	*YWHAB*	3.71
Integrin-linked protein kinase	*ILK*	3.67
EMILIN-1	*EMILIN1*	3.63
Synaptojanin-1	*SYNJ1*	3.62
Annexin A2	*ANXA2*	3.54
AP-2 complex subunit mu	*AP2M1*	3.48
Fermitin family homolog 2	*FERMT2*	3.46
Neprilysin	*MME*	3.42
Myosin-9	*MYH9*	3.41
Palladin	*PALLD*	3.41
Integrin alpha-3	*ITGA3*	3.39
Myoferlin	*MYOF*	3.27
Cytoplasmic dynein 1 light intermediate chain 1	*DYNC1LI1*	3.23
Spectrin beta chain, non-erythrocytic 1	*SPTBN1*	3.23
Gamma-adducin	*ADD3*	3.19
Echinoderm microtubule-associated protein-like 4	*EML4*	3.16
Protein phosphatase methylesterase 1	*PPME1*	3.14
AP-3 complex subunit delta-1	*AP3D1*	3.03
Tubulin beta chain	*TUBB*	2.95
Tubulin alpha-1A chain	*TUBA1A*	2.91
Cadherin-11	*CDH11*	2.89
Glypican-4	*GPC4*	2.88
Filamin-B	*FLNB*	2.86
Myosin-10	*MYH10*	2.86
Fibronectin	*FN1*	2.83
Alpha-actinin-4	*ACTN4*	2.81
Plectin	*PLEC*	2.76
Annexin A7	*ANXA7*	2.74
Integrin alpha-2	*ITGA2*	2.72
EH domain-containing protein 2	*EHD2*	2.71
AP-1 complex subunit mu-1	*AP1M1*	2.71
Epidermal growth factor receptor kinase substrate 8	*EPS8*	2.69
Tropomyosin alpha-1 chain	*TPM1*	2.68
Filamin-C	*FLNC*	2.61
14-3-3 protein epsilon	*YWHAE*	2.6
Kinesin-1 heavy chain	*KIF5B*	2.56
Integrin alpha-V	*ITGAV*	2.55
Calponin-3	*CNN3*	2.45
Filamin-A	*FLNA*	2.45

Importin subunit beta-1	*KPNB1*	2.39
PDZ and LIM domain protein 7	*PDLIM7*	2.38
Dynactin subunit 1	*DCTN1*	2.35
Tropomodulin-3	*TMOD3*	2.34
Spectrin alpha chain, non-erythrocytic 1	*SPTAN1*	2.34
Coatomer subunit alpha	*COPA*	2.34
LIM domain only protein 7	*LMO7*	2.33
Ankycorbin	*RAI14*	2.32
F-actin-capping protein subunit beta	*CAPZB*	2.31
AP-3 complex subunit beta-1	*AP3B1*	2.29
Cullin-4B	*CUL4B*	2.27
Talin-1	*TLN1*	2.24
Voltage-dependent anion-selective channel protein 3	*VDAC3*	2.2
Integrin beta-1	*ITGB1*	2.16
F-actin-capping protein subunit alpha-1	*CAPZA1*	2.15
Vinculin	*VCL*	2.1

The indicated proteins showed a consistent minimum 2-fold increase in the SEPT9 co- immunopreciptated samples compared to IgG controls using lysates derived from two different strains of human TM cells.

Supplemental Table 4. Details of antibodies used in immunoblotting (IB) and immunofluorescence (IF) analyses:

Antibodies	Cat. No.	Source	Dilution (IB)	Dilution (IF)
Septin9 Rabbit Polyclonal	HPA042564	Millipore Sigma, St. Louis, MO, USA	1:1000	1:200
Septin11 Rabbit polyclonal	440 003	Synaptic Systems, Goettingen, Germany	1:1000	1:200
Septin7 Rabbit polyclonal	HPA029524	Millipore Sigma, St. Louis, MO,	1:1000	1:200
Septin2 Rabbit polyclonal	HPA018481	Millipore Sigma, St. Louis, MO,	1:1000	1:200
α-Actinin1 Mouse Monoclonal	SAB4200813	Millipore Sigma, St. Louis, MO,	1:1000	
Phalloidin–Tetramethyl rhodamine B isothiocyanate (TRITC)	P1951	Millipore Sigma, St. Louis, MO,		1:500
Alexa Fluor™ 647 Phalloidin	A22287	Thermo Fisher Scientific. Eugene, OR		1:400
Vinculin mouse monoclonal	V9131	Sigma/Aldrich, St. Louis, MO		1:200
p-FAK (Tyr397) Rabbit monoclonal	700255	Thermo Fisher Scientific, Waltham, MA.	1:1000	
p-Paxillin(Tyr118) Rabbit polyclonal	2541	Cell Signaling Technology, Inc, Danvers, MA	1:1000	
p-Myosin Light Chain 2 (Thr18/Ser19) Rabbit polyclonal	3674	Cell Signaling Technology, Inc, Danvers, MA	1:1000	
JCAD Rabbit polyclonal	NBP1-0941	Novus Biologicals USA, Littleton, CO		1:200
p-MYPT1 (Thr696) Rabbit Polyclonal	ABS45	Millipore Sigma, St. Louis, MO	1:1000	
α-smooth muscle actin- Cy3 conjugated	C6198	Millipore Sigma, and St. Louis, MO		1:500
Collagen1V α1 Rabbit polyclonal	ab189408	abcam, Waltham, Boston, MA		1:100
COL1A1 Rabbit Rabbit polyclonal	72026	Cell Signaling Technologies, Inc., Danvers, MA	1:1000	
GAPDH Mouse Monoclonal	60004-1	Proteintech Group, Chicago, IL	1:5000	
Fibronectin Rabbit polyclonal	-	Harold P. Erickson, PhD, Cell Biology, Duke University, Durham, NC.		1:8000
Hoechst 33258, Pentahydrate (bis-benzimide)	H21491	Thermo Fisher Scientific. Eugene, OR		1:1000
Iba-1 Rabbit polyclonal	019-19741	Fujifilm Wako Chemicals. VA		1:1000
APC anti-mouse F4/80	123115	Biolegend, San Diego, CA		1:500

Secondary antibodies for immunofluorescence analyses:

Antibodies	Cat. No.	Source	Dilution
Alexa Fluor^{TM} 488 goat anti-Rabbit IgG	A11077	Invitrogen / Thermo Fisher Scientific. Rockford, IL	1:200
Alexa Fluor^{TM} 568 goat anti-mouse IgG	A11004	Invitrogen / Thermo Fisher Scientific. Rockford, IL	1:200

Secondary antibodies for immunoblot analyses:

Antibodies	Cat. No.	Source	Dilution
Peroxidase AffiniPure Goat Anti-Rabbit IgG (H+L)	111-035-144	Jackson ImmunoResearch Inc, West Grove, PA	1:5000
Goat anti-Mouse IgG (H+L) Secondary Antibody, HRP	31430	Invitrogen / Thermo Fisher Scientific. Rockford, IL	1:5000
