# Supplementary material for: Dysregulation of septin cytoskeletal organization in the trabecular meshwork contributes to ocular hypertension
Source: JCI Insight. 2024 Dec 6;9(23):e179468. doi: 10.1172/jci.insight.179468 (PMC11623952; doi:10.1172/jci.insight.179468)

Immunoblots used for the manuscript figures

Figure. 1A

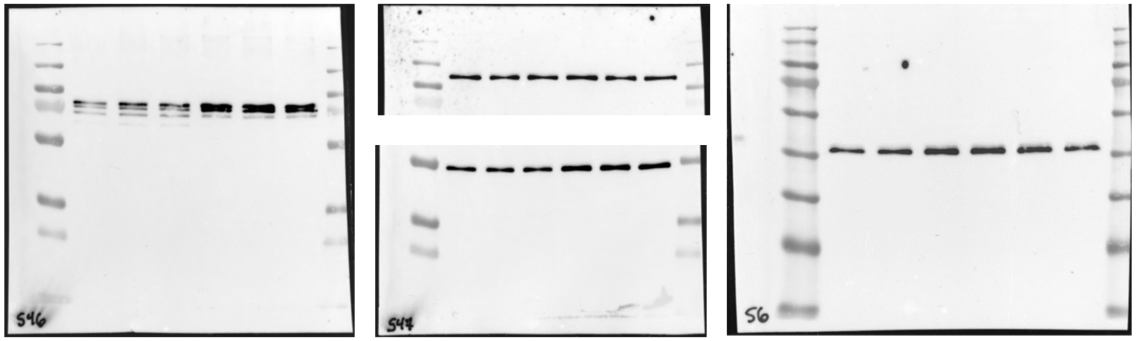

Figure. 1C

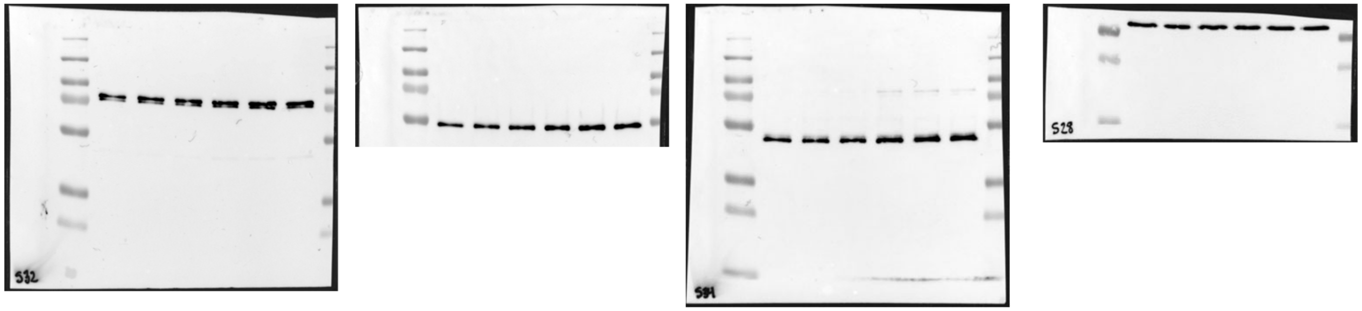

Figure. 1E

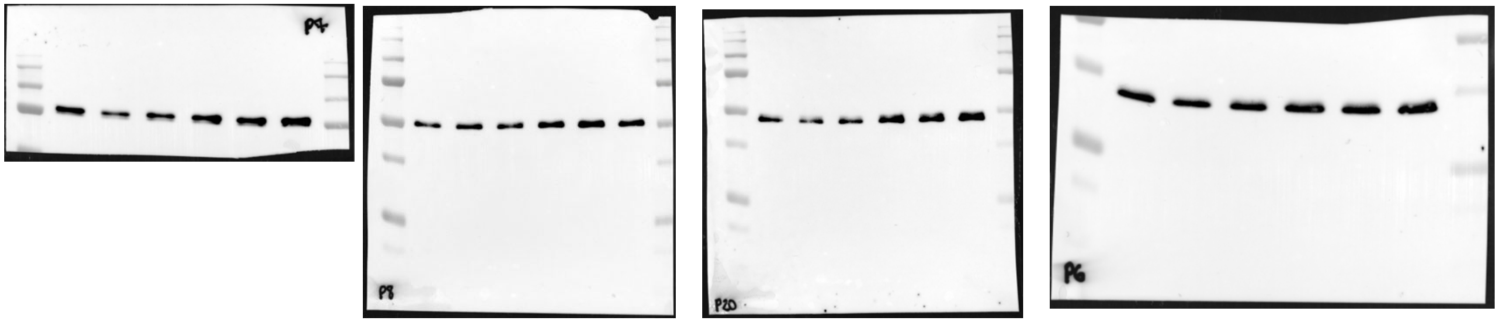

Figure.3D

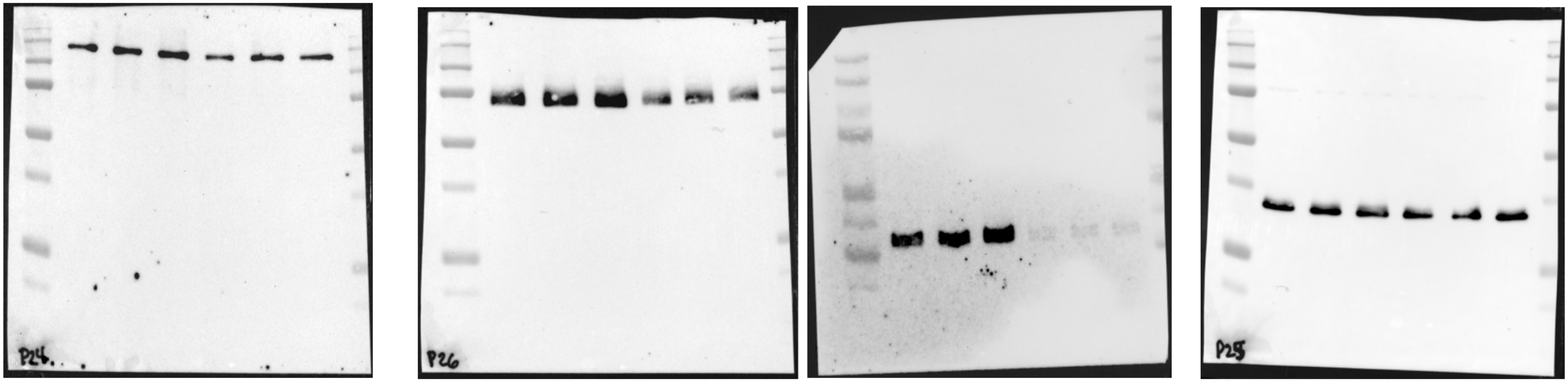

Figure. 4B

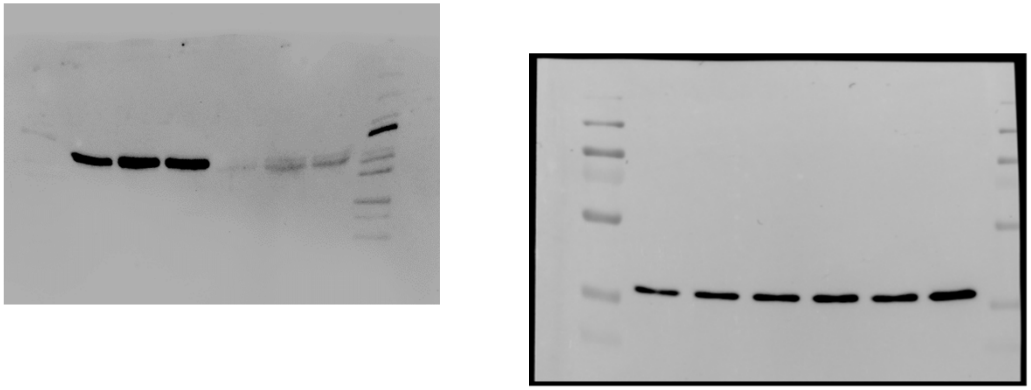

Figure. 4F

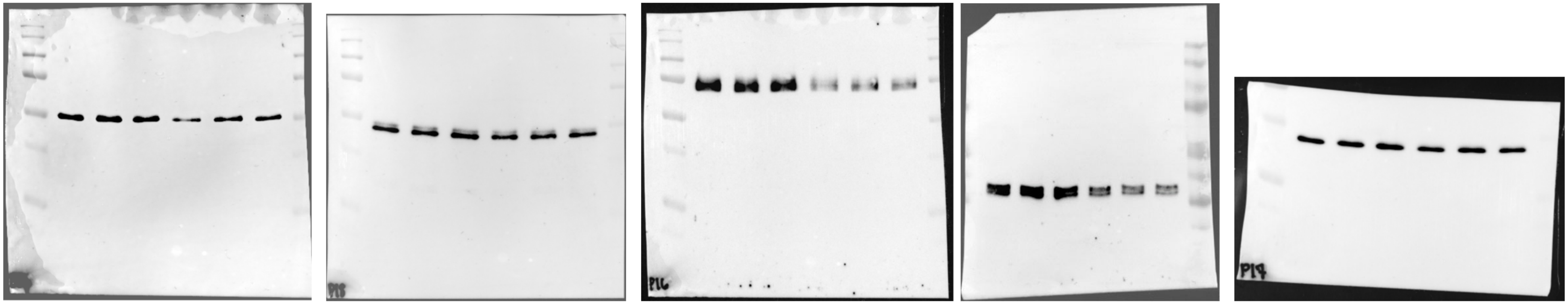

Figure. 5C

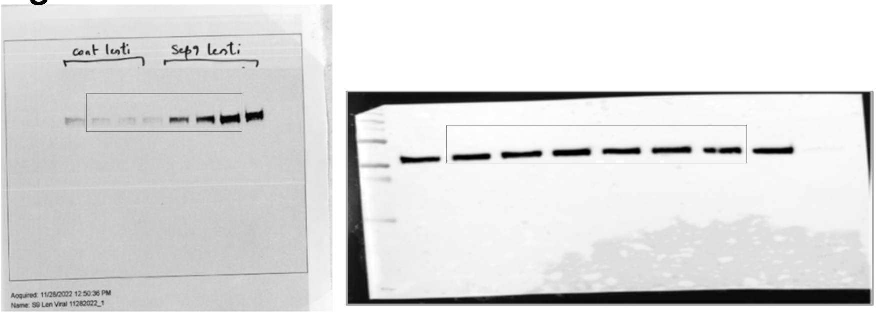

Figure. 5G

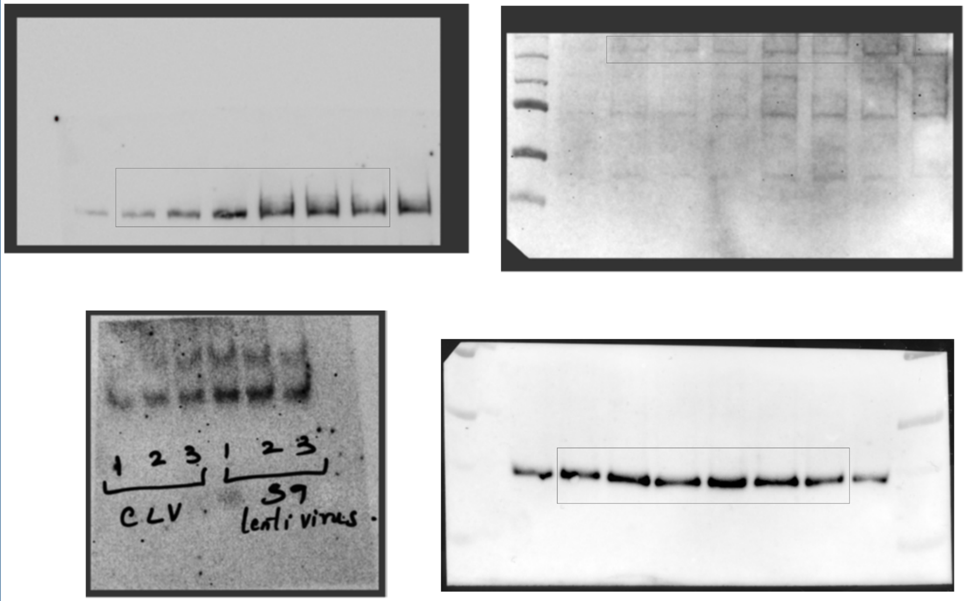

Figure. 5D

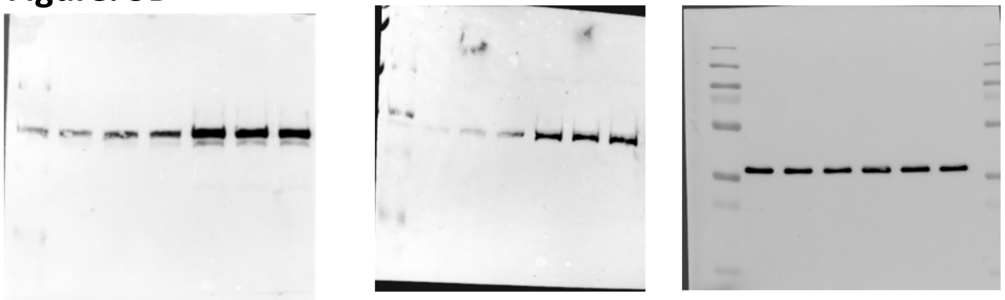

Figure. 5I

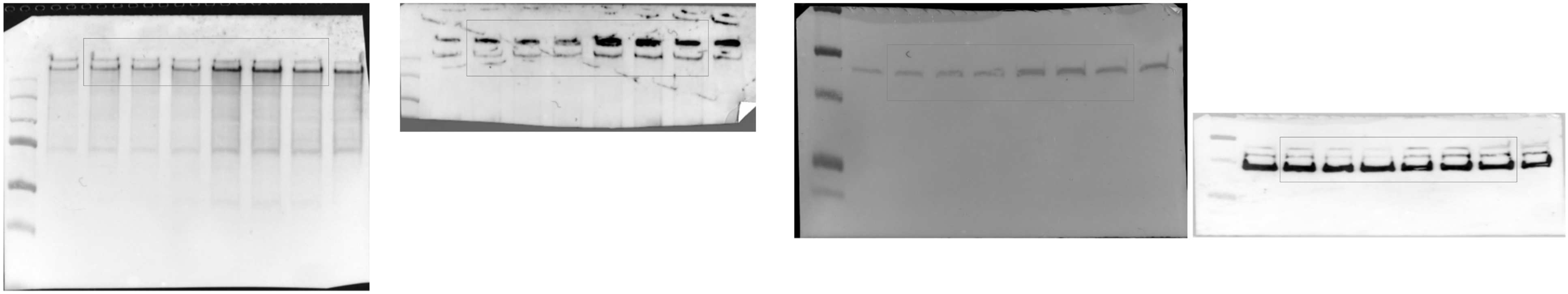

Supplemental Figure 1

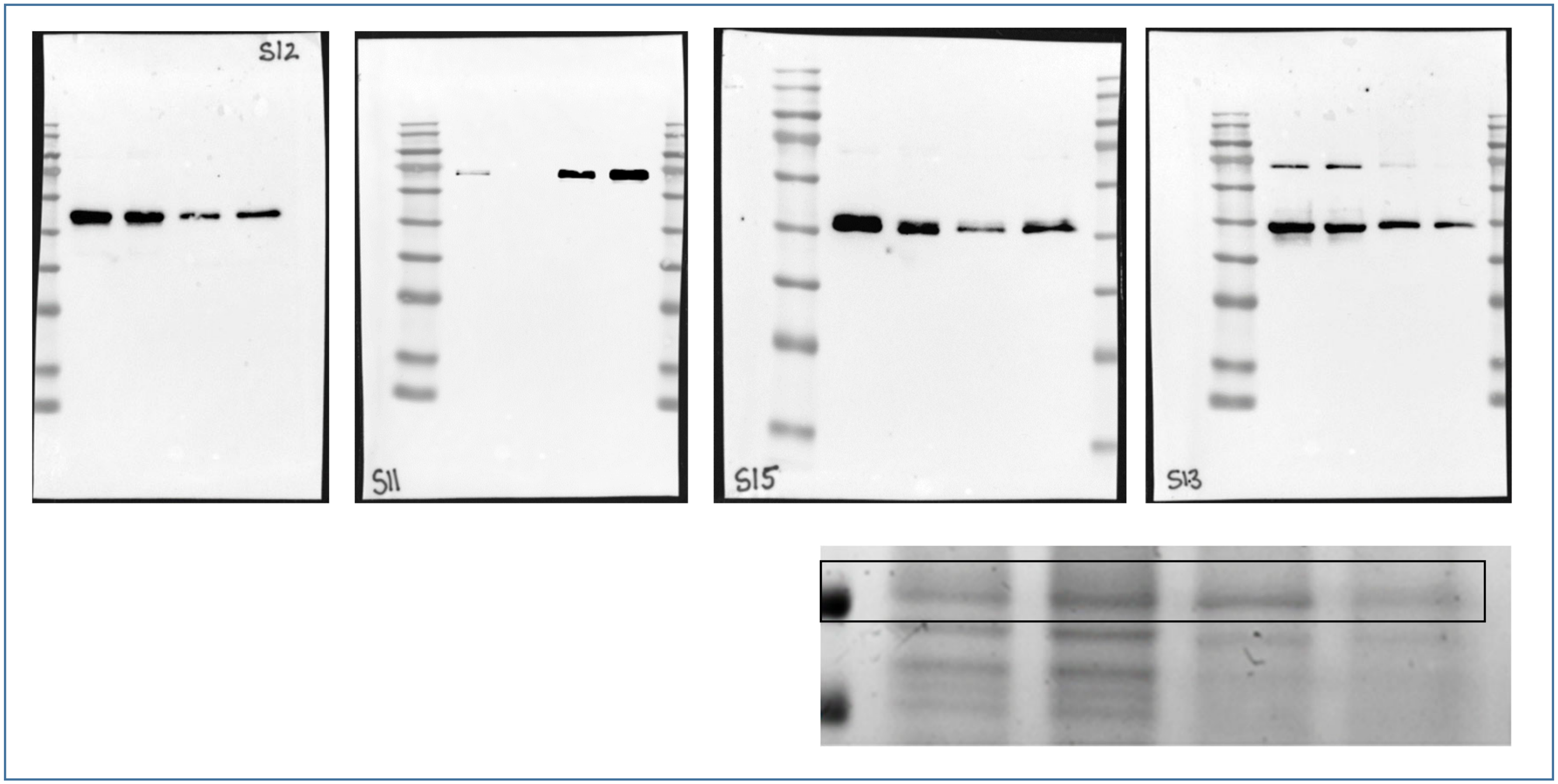

Supplement: Unedited blot and gel images [file jciinsight-9-179468-s107.pdf]
